# Superior TRAIL gene expression and cancer cell apoptosis mediated by highly branched-linear poly(β-amino ester)s

**DOI:** 10.1186/s12951-023-02169-7

**Published:** 2023-10-28

**Authors:** Yitong Zhao, Tao Bo, Chenfei Wang, Dingjin Yao, Chaolan Pan, Weiyi Xu, Hao Zhou, Ming Li, Si Zhang

**Affiliations:** 1 School of Medicine, Anhui University of Science and Technology, 232000 Huainan, Anhui China; 2 NHC Key Laboratory of Glycoconjugate Research, Department of Biochemistry and Molecular Biology, School of Basic Medical Sciences, Fudan University, 200032 Shanghai, China; 3 Department of Dermatology, Children’s Hospital of Fudan University, National Children’s Medical Center, 201102 Shanghai, China; 4 State Key Laboratory of Medicinal Chemical Biology, Tianjin Key Laboratory of Protein Science, College of Life Sciences, Nankai University, 300071 Tianjin, China

**Keywords:** Cancer therapy, *TRAIL* gene, Branched-linear poly(β-amino ester)s, Cell apoptosis

## Abstract

Extensive efforts have been dedicated to enhancing the expression of tumor necrosis factor-related apoptosis-inducing ligand (TRAIL) in cancer cells for the development of effective cancer treatments. However, highly safe and efficient delivery of *TRAIL* gene remains a significant challenge, especially using cationic polymers. Here, a series of highly branched-linear poly(β-amino ester)s (H-LPAEs) are developed through a unique oligomer branching strategy. H-LPAEs exhibit a more uniform distribution of linear segments and branching units, leading to excellent DNA condensation and favorable physicochemical properties of H-LPAE/DNA polyplexes. In SW1353 and BMSC cells, the optimized H-LPAEs, H-LPAE_B4−S5−TMPTA_, achieves superior gene transfection efficiency of 58.0% and 33.4%, which were 2.5-fold and 2.0-fold higher than that of the leading commercial gene transfection reagent, Lipofectamine 3000. Excitingly, H-LPAE_B4−S5−TMPTA_ mediated 56.7% and 28.1% cell apoptosis in HepG2 cells and HeLa cells highlighting its potential application in cancer gene therapy. In addition, locally administered H-LPAE_B4−S5−TMPTA_ delivered *TRAIL* DNA to HepG2 xenograft tumors and inhibited tumor growth in vivo. This study not only proposes a novel strategy for synthesizing poly(β-amino ester)s with a unique branched-linear topology but also identifies a promising candidate for highly efficient TRAIL gene transfection.

**Supplementary Information:**

The online version contains supplementary material available at 10.1186/s12951-023-02169-7.

## Introduction

Tumor necrosis factor (TNF)-related apoptosis-inducing ligand (TRAIL) is a highly promising antitumor agent that specifically induces tumor cell apoptosis without killing normal cells and binding with apoptosis receptors [1–3]. However, the short half-life of TRAIL in blood circulation and its poor in vivo stability greatly limited its anti-tumor effects and therapeutic efficacy [4, 5]. Gene therapy, which involves delivering functional *TRAIL* genes into cancer cells or tissues to manipulate the stable expression of TRAIL protein, holds significant potential for the treatment of advanced cancer [6, 7]. To date, the development of an effective and non-immunogenic vector for *TRAIL* gene delivery remains a challenge to accelerate the translation of cancer gene therapy from the laboratory to clinical applications.

In the last few years, great efforts have been dedicated to the development various promising non-viral gene vectors. Among them, cationic polymers have emerged as highly promising candidates due to their simplicity, ease of large-scale synthesis, tailored structure, low cytotoxicity, and non-immunogenicity [8, 9]. Topological structure has significant effects on the gene transfection performance of cationic polymers. Notably, in 2015, our group successfully synthesized highly branched poly(β-amino ester)s (HPAEs) through an “A2 + B3 + C2” Michael addition strategy for efficient DNA delivery both in vitro and in vivo [10, 11]. These HPAEs possessed tailorable composition, structure, molecular weight (MW), biodegradability, high stability, and excellent biocompatibility [11]. The one-pot copolymerization of amine (A2), triacrylate (B3), and diacrylate (C2) not only delayed gelation during the polymerization process but also resulted in three-dimensional (3D) topology and multiple terminal groups in the HPAEs [12, 13]. Through optimization, the HPAE exhibited showed up to 8521-fold enhancement in gene transfection efficiency compared to the corresponding linear poly(β-amino ester)s (LPAEs) in vitro [10]. More importantly, HPAEs could efficiently deliver *COL7A1* plasmids to restore high-level collagen VII (C7) expression in recessive dystrophic epidermolysis bullosa (RDEB) knockout and grafting mouse models [10, 14, 15].

Nevertheless, utilizing the generalizable one-pot Michael addition strategy, all HPAEs exhibited broad molecular weight distribution (MWD) with the polydispersity index (*Đ*) values up to 12.0 [12, 16–18], leaded to poor homogeneity and stability, presenting additional challenges in understanding the mechanistic impact of MW on their gene transfection activity, which was not conducive for nano-sized polyplexes to efficiently overcome various extracellular and intracellular barriers, as well as clinical approval of the products. In 2023, we employed a step-by-step precipitation strategy to fractionate HPAEs with a similar composition and relatively narrow *Đ* from the crude polymer and preliminarily compared their gene transfection performance in vitro [16]. However, the step-by-step precipitation strategy requires a large volume of solvent and does not address the root cause of the broad MWD. Therefore, we hypothesized that redesigning the polymerization strategy based on the reaction mechanism could regulate the MWD of HPAEs and enhance transfection efficiency.

Herein, we developed a new structure of linear-branched poly(β-amino ester)s (H-LPAEs) with a more uniform distribution of linear segments and branching units through the linear oligomer combination branched strategy, and evaluated its *TRAIL* gene delivery profile in cancer cell lines. We further optimized the DNA transfection parameters and demonstrated that the optimized HPAEs are effective vehicles for delivering DNA to diverse cancer cell types. To further explain the effect of topology on both the biological properties of the polyplexes and the transfection mechanism, we characterized the DNA binding and condensation, polyplex size and zeta potential, microscopic morphology, cellular uptake, and endosomal escape. Excitingly, the tumor cell apoptosis results revealed that H-LPAE_B4−S5−TMPTA_ mediated 56.7% and 28.1% cell apoptosis in HepG2 cells and HeLa cells highlighting its potential application in cancer gene therapy. Therefore, the rational design and optimization of H-LPAEs may further facilitate the clinical applications of non-viral gene vectors and advance our understanding of cancer gene therapy.

## Materials and methods

### Materials

4-amino-1-butanol (S4, Energy Chemical, 97%), 5-amino-1-pentanol (S5, TCI, 95%), 1,4-butanediol diacrylate (B4, Aladdin, > 90%), trimethylolpropane triacrylate (TMPTA, Macklin, 95%), pentaerythritol tetraacrylate (PET4A, Macklin, 95%), 1-(3-aminopropyl)-4-methylpiperazine (E7, Energy Chemical, 97%), 3,6,9-trioxaundecamethylenediamine (122, adamas, 98%), lithium bromide (LiBr, Aladdin, 99.9%), dimethyl sulfoxide (DMSO, Macklin, 99.9%), dimethylformamide (DMF, J&K Scientific, 99.9%), diethyl ether (Sinopharm Chemical Reagent, 99%), and deuterated chloroform (CDCl_3_, Sigma-Aldrich, 99.9%) were purchased without further purification. PicoGreen dsDNA Quantitation Reagent (Yeasen Biotechnology), AlamarBlue (Yeasen Biotechnology), jetPEI (Polyplus), Lipofectamine 3000 (Thermo Fisher Scientific), AF647-labeled DNA (Thermo Fisher Scientific) were used according to the manufacturer’s protocol. Plasmids coding for green fluorescence protein (pCMV-GFP) were purchased from Aldvron (USA).

### H-LPAEs synthesis and purification

Four H-LPAEs were synthesized *via* a linear oligomer combination branched strategy involving two sequential steps. In the first step, LPAE-ac contained vinyl groups at both ends was synthesized *via* the Michael addition strategy. All monomers were dissolved in DMSO (500 mg/mL) according to feed ratios (Table S1, and S2, Supplementary Materials), and the mixture was reacted at 90 °C under magnetic stirring. The growth of *M*_w_, *M*_n_, and *Đ* during the polymerization process was monitored using GPC. Once the *M*_w_ reached the range of 4.5 kg/mol-10 kg/mol, the reaction was halted by cooling to room temperature (RT) and then diluted with DMSO. To end-cap the polymer, a second amine, 122 or E7, was added to react for an additional 48 h at RT. For purification, the crude products were precipitated with excess diethyl ether three times and then dried in a vacuum oven at RT. In the second step, tri-acrylates and tetra-acrylates were added to react with LPAEs oligomers to generate H-LPAEs at an LPAEs oligomer concentration of 300 mg/ml at 90 °C (Table S3, S4, S5, and S6, Supplementary Materials). Similarly, GPC further was used to monitor the growth of *M*_w_, *M*_n_, and *Đ* during the polymerization process. When the *M*_w_ reached the range of 15.0 kg/mol-20.0 kg/mol, the reaction was halted by cooling to RT and then diluting it with DMSO to a final concentration of 100 mg/mL. A second amine, 122 or E7, was added to the end-cap of the polymer. The purification of H-LPAE was conducted following the aforementioned procedure.

### Gel permeation chromatography (GPC) measurements

To determine the measure the weight average MW (*M*_w_), number average MW (*M*_n_) and polydispersity index (*Đ*) values of polymers, An Agilent 1260 infinity II GPC equipped with a refractive index (RI) detector was utilized. For analysis, 10.0 mg of the polymer samples were dissolved in 1 mL of DMF containing with 0.1% LiBr, then the sample is vigorously vortexed for 1–3 min, and filtered through a 0.22 μm filter. DMF containing 0.1% LiBr was used to eluted from GPC columns (PolarGel-M Gard, 50 mm × 7.5 mm, and PolarGel-M, 300 mm × 7.5 mm, two in series) at a flow rate of 1 mL/min. The column temperature was maintained at 50 °C. The GPC column was calibrated using linear poly(methyl methacrylate) (PMMA) standards with three defined MW.

### Nuclear magnetic resonance (NMR) measurements

^1^ H NMR spectroscopy was employed to verify the chemical composition, purity, and structure of the H-LPAEs. NMR measurements were conducted on a Varian Inova 400 MHz Spectrometer (Bruker, Switzerland). The polymers were then dissolved in CDCl_3_ for analysis. Chemical shifts of the sample were reported in parts per million (ppm) relative to CDCl_3_.

### Polyplex formulation

The polymers were initially dissolved in DMSO to form a stock solution with a concentration of 100 mg/mL. The polymer stock solution and DNA were separately diluted in 10 µL of sodium acetate solution (0.025 M, pH = 5.2) according to the desired polymer/DNA weight ratio (w/w). Subsequently, the polymer solution was then added to the DNA solution and vigorously vortexed for 25–30 s at high speed to facilitate thorough mixing. The mixed solution was left undisturbed for 25 min to form polyplexes.

### DNA binding affinity of HPAEs

The DNA binding affinity of the polymers was measured using the Picogreen assay. Polyplexes were prepared with 1.5 µg of DNA as described above, and an equivalent volume of PicoGreen solution was added and incubated for another 5 min (16 µL PicoGreen diluted to 3.04 mL of sodium acetate (0.025 M, pH = 5.2). The mixed solution was aliquoted three times and diluted with 200 µL of serum-free Dulbecco’s Modified Eagle Medium (DMEM) in a black 96-well plate. A plate reader (Synergy Hybrid H1, Biotek) with excitation at 490 nm and emission at 535 nm was used to measure the fluorescence. Samples with naked DNA were used as negative controls and samples without DNA were used as blanks. The DNA-binding affinity of the polymers was calculated as follows: DNA-binding affinity (%) = 1-(*F*_Sample_-*F*_Blank_)/ (*F*_DNA_-*F*_Blank_). All measurements were repeated at least three times.

### Size and zeta potential of polyplexes

Each polyplex was prepared with 1 µg of DNA according to the polymer/DNA w/w ratio, as described above, and then diluted in deionized water to 1.0 mL. A Malvern Instruments Zetasizer (Nano ZSE) with a scattering angle of 173 ° was used to measure the sizes and zeta potentials of the polyplexes. All measurements were repeated at least three times.

### The morphology of polyplexes

For each sample preparation, 0.5 µg of DNA was used. The polyplexes were prepared at a w/w ratio of 20:1, as described above. After the polyplexes were incubated for 15–25 min, deionized water was used to remove inorganic salts. This process is repeated three times. Deionized water (100 µL) was then added to disperse the polyplexes. Five microliters of the dispersed polyplexes were dropped onto a copper-coated grid and freeze-dried immediately for 30 min. The morphology of the polyplexes was observed by transmission electron microscopy (TEM).

### Cell culture

HeLa, HepG2, SW1353, and RSC96 cells were cultured in DMEM containing 1% penicillin/streptomycin (P/S) and 10% FBS. BMSC cells were cultured in α-MEM (containing 1% penicillin/streptomycin (P/S) and 10% FBS). All cells were cultured in a humid incubator (37 °C, 5% CO_2_) under standard culture conditions.

### DNA transfection efficiency evaluation

Cells were seeded in 96-well plates at a density of 2.0 × 10^4^ cells/well and cultured to 70–80% confluence. For each well, 0.5 µg of DNA (GFP plasmids) was used, and polyplexes were prepared at polymer to DNA weight ratios of 20:1, 40:1 and 60:1, respectively. The polyplex solution was then diluted to 100 µL with DMEM containing 1% P/S and 10% FBS. The cell supernatant was removed from the cell culture plate, 100 µL of polyplex-containing DMEM was added, and the cells were cultured for another 48 h. Transfected cells were evaluated and GFP expression was visualized using a fluorescence microscope (Olympus CKX53). The cells were then collected and washed with PBS, and at least 5,000 cells were counted for each sample. A flow cytometer (ACEA NovoCyte) was used to quantitatively analyze transfection efficiency.

### Cell viability evaluation

The cytotoxicity of the polyplexes after transfection was measured using the alamarBlue assay. Briefly, the cell culture medium was removed, and the cells were washed twice with PBS. Next, 100 µL of diluted alamarBlue solution (10%) was added, and the cells were incubated for another 0.5 to 1 h. The fluorescence intensity of the supernatant was measured using a microplate reader (Synergy Hybrid H1, Biotek) at excitation and emission wavelengths of 530 and 590 nm, respectively. Cells not transfected with polyplexes were used as controls and were defined as having 100% viability. Each group was measured at least thrice.

### Cancer cell apoptosis assay

Cancer cells were seeded in 96-well plates at a density of 1.0 × 10^4^ cells/well and cultured until 60–70% confluence. For each well, 0.5 µg of DNA (TRAIL-GFP plasmids) was utilized, and polyplexes were prepared at a w/w ratio of 20:1, 40:1 and 60:1. The polyplex solution was diluted to 100 µL using DMEM. After 4 h of incubation, the supernatant was removed from the cell culture plate. Polyplex-containing 10% DMEM (100 µL) was added, and the cells were cultured for another 44 h. Transfected cells were evaluated and GFP expression was visualized using a fluorescence microscope (Olympus CKX53). Apoptosis was evaluated using the AnnexinV-PE/7-AAD (MA0429, Promega) Apoptosis Detection Kit and flow cytometry. Briefly, after appropriate treatment, the cells were collected using EDTA-free trypsin, washed twice with PBS, and stained with 5 µL of Annexin V-PE and 5 µL of 7-ADD for 20 min at RT in the dark prior to flow cytometric analysis. Untreated cells were used as a negative control, and cells single-stained with Annexin V-PE and 7-ADD were used as gate criteria [1–3]. Each group was measured at least thrice.

### In vivo anti-tumor efficacy

BALB/c-nude mice (4 weeks, male) were purchased from Shanghai SLAC Laboratory Animal Co., Ltd (Shanghai, China) and maintained under pathogen-free conditions. The xenograft tumor model was established via subcutaneous inoculation of HepG2 cells (5 × 10^6^) in the flank of BALB/c-nude mice. H-LPAE_B4−S5−TMPTA_ were synthesized with empty or TRAIL plasmid in pH 7.4 sodium acetate at a DNA dose of 0.2 µg/µL and stored at -80℃. When the tumor volume reached ~ 50 mm^3^, mice were randomly divided into three groups (3 mice per group) and received intratumoral injections of H-LPAE_B4−S5−TMPTA_ or intravenous administration of vorinostat (150 µM). Every 4 days, the drug was administered and the tumor volume was measured (V = 0.5 × length × width^2^). On day 20, the mice were sacrificed and tumors were harvested.

### Western blot

Total protein was extracted from tissues after using RIPA lysis and centrifugation, and was boiled at 95℃ for 10 min. Protein was transferred to the PVDF membrane after electrophoretic separation. The membrane was then incubated with the diluted primary antibodies: Cleaved Caspase 3 (Proteintech, 25128-1-AP, 1:1000), Caspase 3 (Proteintech, 19677-1-AP, 1:10000), Caspase 8 (Proteintech, 13423-1-AP, 1:800), Cleaved Caspase 8 (CST, 8592 S, 1:1000), TRAIL (CST, 3219 S, 1:1000) overnight at 4℃ and 1:10000 secondary antibodies (Proteintech, SA00001-1/, SA00001-2, 1:10000) for 1 h the next day. Chemiluminescence was performed for the results.

### Statistical analysis

The student’s t-test was used to examine the cell viability, mean fluorescence intensity and apoptosis efficiency; results are reported as average values ± standard deviation (SD). The average values and SD were calculated from at least three independent experimental results. The *p* values < 0.05 were considered statistically significant.

## Result and discussion

**Fig. 1:**
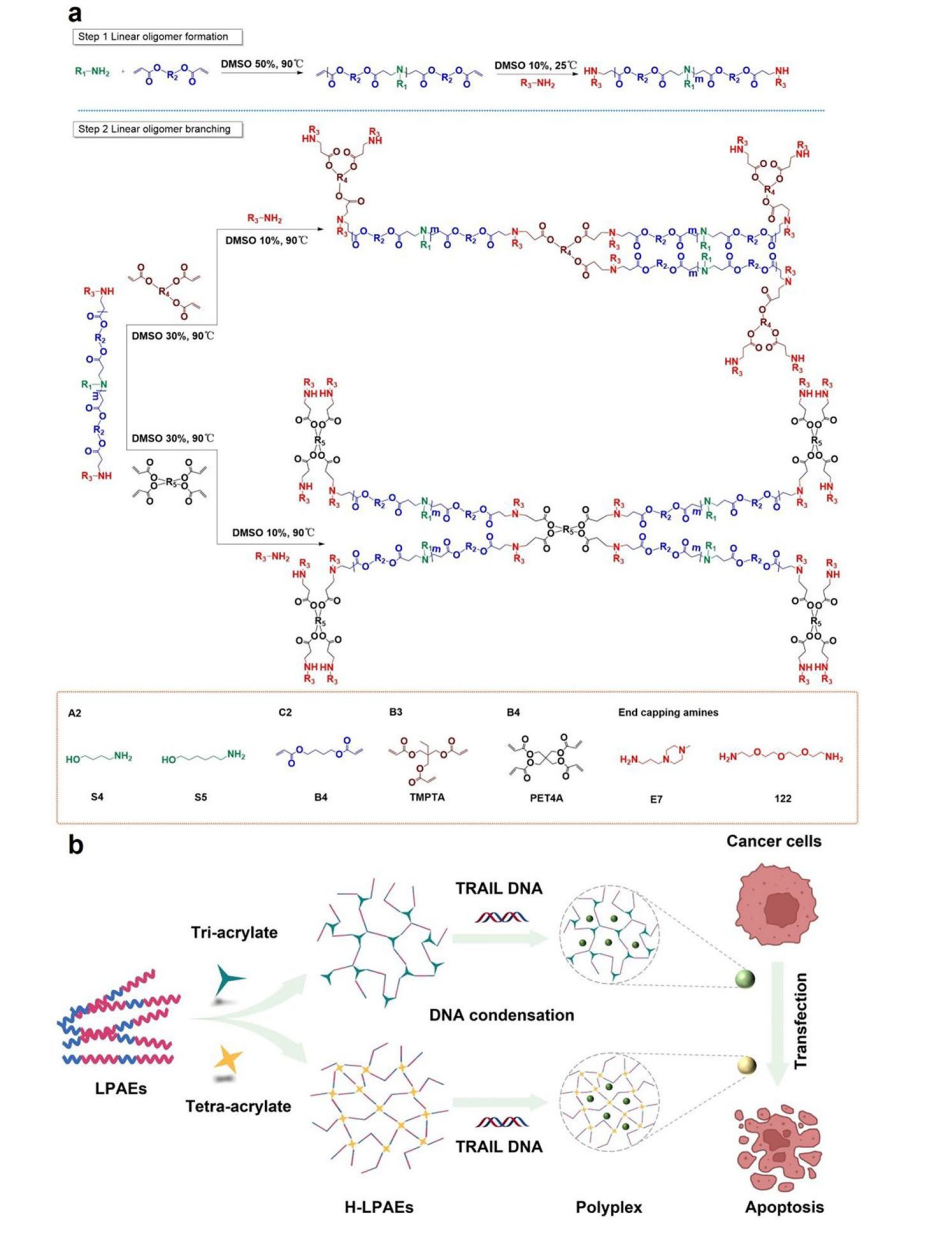
H-LPAE-based gene delivery system for the *TRAIL* gene transfection of various cancer cells. **(a)** Schematic illustration of the synthesis of H-LPAEs through the two sequential linear oligomers combination branched strategies; **(b)** H-LPAEs condenses *TRAIL* DNA to formulate nano-sized polyplexes and induced tumor cell apoptosis for various cancer cells

### H-LPAEs synthesis and characterization

**Fig. 2:**
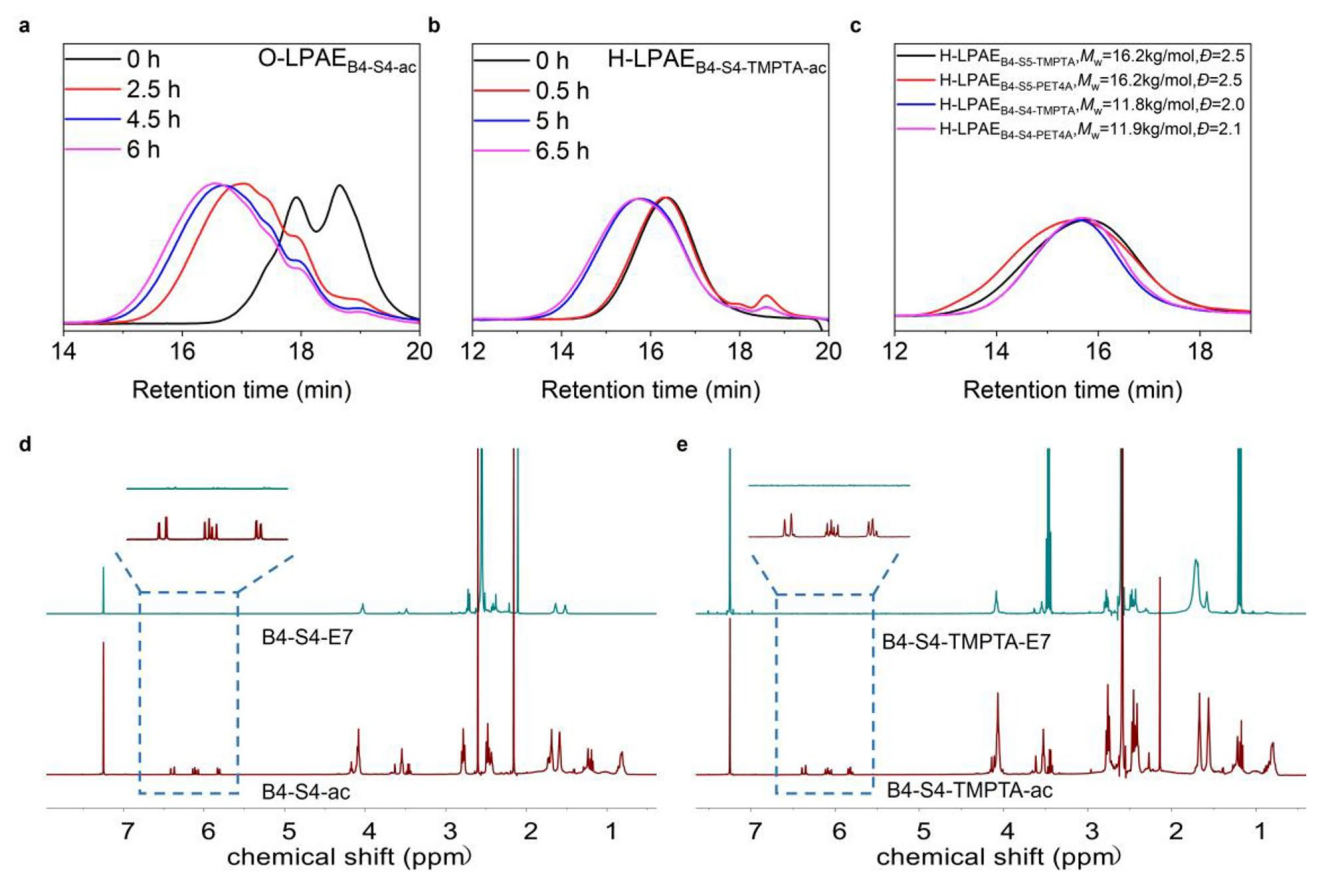
Synthesis and characterization of H-LPAEs *via* the linear oligomer combination branched strategy. **(a)** GPC traces of O-LPAE_B4−S4−ac_ during the polymerization process. **(b)** GPC traces of H-LPAE_B4−S4−TMPTA−ac_ during the polymerization process. **(c)** GPC traces of H-LPAEs different compositions. **(d)** and **(e)**^1^ H NMR spectra of LPAE_B4−S4_ and H-LPAE_B4−S4−TMPTA_ before **(d)** and after **(e)** end-capping and purification

Previous enormous efforts and research have demonstrated that many HPAEs with different compositions and structures have been successfully synthesized and screened for gene transfection [10, 14, 15]. Among them, HC32 and H447 have been identified as the most effective candidates both in vitro and in vivo [13, 19]. However, due to the iterative step-growth polymerization and branching process, these HPAEs exhibited a broad MWD and inhomogeneous distribution of linear segments and branching units, which imposed extra challenges in the mechanistic understanding of the effect of MW on their gene transfection activity. To address this issue, we developed a novel strategy called the linear oligomer combination branched approach (Fig. 1), which enabled the synthesis of four H-LPAEs with a more uniform distribution of linear segments and branching units. This strategy involved two sequential steps. In the first step, LPAE-ac with vinyl groups on both ends was synthesized using a Michael addition strategy, employing a stoichiometric ratio of acrylates to amine of 1.2:1.0 and a total monomer concentration of 500 mg/ml. Figure 2a and Table S7 illustrated that at 2.5 h, the MW of polymer is around 3.1 kg/mol with a narrow *Đ* of 1.4. As the polymerization progressed, both the MW and *Đ* increased rapidly to 4.0 kg/mol and 1.6, respectively. The polymerization was then halted, and E7 was added to end-cap the polymer at RT for 48 h. After purification with diethyl ether, the MW and *Đ* of the product LPAEs were 5.3 kg/mol and 1.5. respectively. ^1^ H NMR spectroscopy further confirmed the successful copolymerization of the A2 and B2 monomers **(**Fig. 2d). In the second step, tri-acrylates and tetra-acrylates were added to react with the LPAEs oligomers to generate H-LPAEs at an LPAEs oligomer concentration of 300 mg/mL.

As the branched monomers were reacted with the pre-synthesized linear oligomers, thus the synthesized H-LPAE exhibited relatively narrow *Đ*. As shown in Fig. 2b, in the earlier stage of polymerization, due to the high concentration of branched monomers, branched monomers are linked to multiple oligomers, resulting in slower polymerization kinetics as indicated by the GPC traces. As the polymerization proceeded, MW and *Đ* of the polymer further increased to 10.2 kg/mol and 1.8, respectively, after 5 h of polymerization (**Table S8**). Polymerization was stopped after 6.5 h of reaction, when MW was approaching 10.8 kg/mol, and E7 was added to end-cap the polymer at RT for 48 h to obtain a polymer without vinyl groups. After purification, H-LPAE_B4−S4−TMPTA_ with MW of 11.8 kg/mol and *Đ* of 2.0 was obtained (Fig. 2c). The ^1^ H NMR analysis revealed that the characteristic peaks of the double bonds of H-LPAE at 5.5-6.0 ppm completely disappeared after end-capping, indicated that H-LPAE_B4−S4−TMPTA_ was successfully synthesized (Fig. 2e). More importantly, three H-LPAEs with different chemical compositions were successfully synthesized using a similar polymerization strategy (Figure S1-S9). In addition, GPC traces suggested that none of the polymerization processes resulted in gelation, further indicating that our proposed sequential branching strategy is well controllable. These findings indicated that our approach can serve as a versatile platform technology for the synthesis of H-LPAEs (Figure S1-S6, and Table S7-S14).

### The physiochemical properties of H-LPAE and formulated polyplexes

**Fig. 3:**
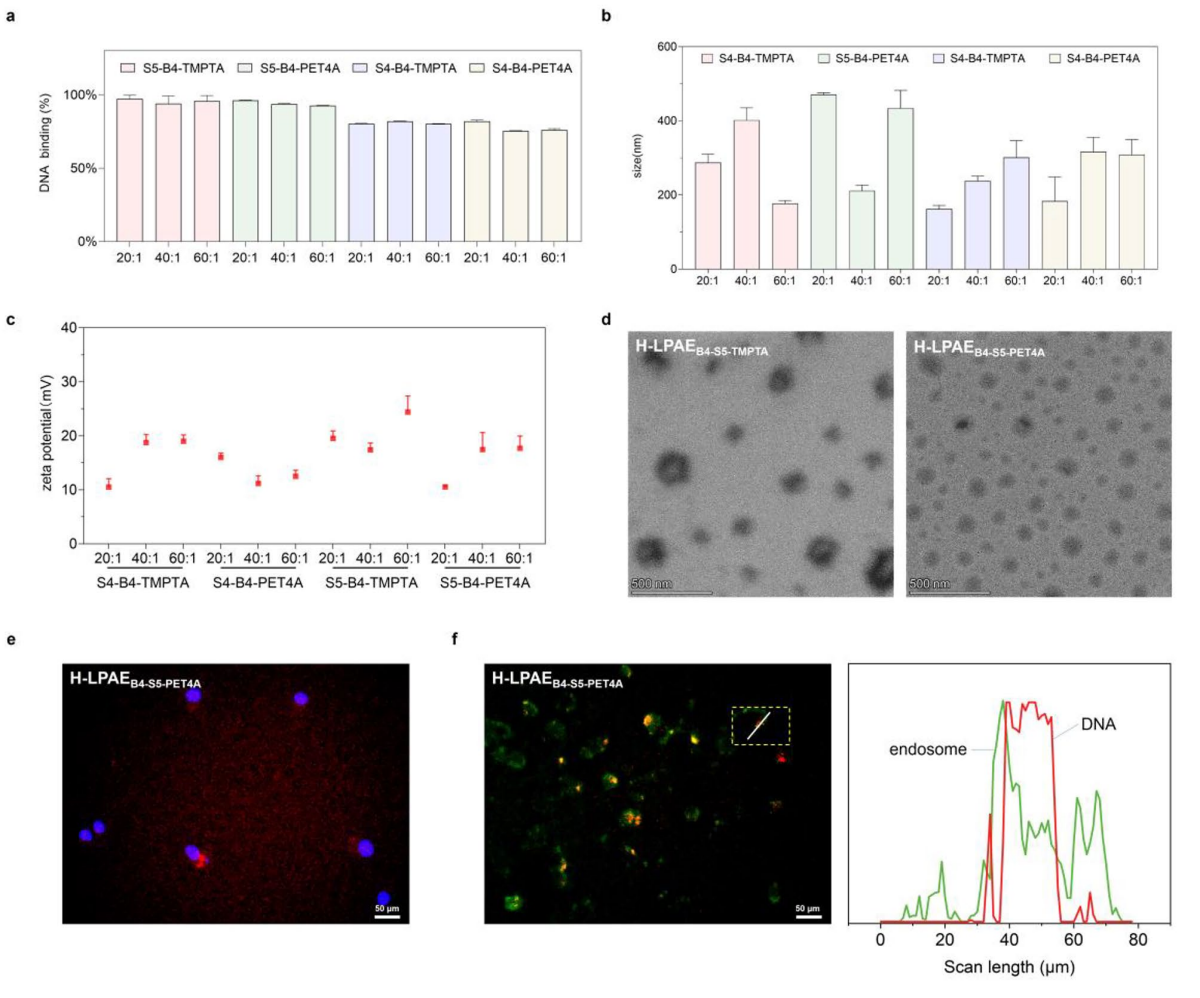
Physicochemical properties of H-LPAE/DNA polyplexes.** (a)** DNA binding affinity measured by PicoGreen assays (n = 3); **(b)** and **(c) **Size **(b) **and Zeta potential **(c)** of polyplexes (n = 3); **(d) **TEM images of two H-LPAEs/DNA polyplexes at a w/w of 20:1. The scale bar is 500 nm. **(e) **Representative cellular uptake images of HeLa cells after incubation with AF647-labeled polyplexes, Scale bars, 50 μm. **(f) **Representative fluorescence colocalization images of H-LPAE_B4−S5−PET4A_/ AF647-DNA polyplexes and acidic endosomes stained with Lyso Tracker Green. Scale bars, 50 μm

In general, cationic polymers with a stronger DNA-binding affinity tend to condense DNA more tightly, resulting in the formation of polyplexes with smaller sizes, higher zeta potentials and stability. These characteristics are favorable to cellular uptake and endosomal escape of polyplexes, ultimately enhancing gene transfection efficiency. Therefore, to further understand the mechanism behind the enhancement in gene transfection performance of H-LPAEs with different components, the DNA-binding affinities of H-LPAE_B4−S5−TMPTA_, H-LPAE_B4−S5−PET4A_, H-LPAE_B4−S4−TMPTA_ and H-LPAE_B4−S4−PET4A_ were determined using the PicoGreen assay. Figure 3a illustrated that all four H-LPAEs exhibited DNA-binding affinities exceeding 75.5%. In contrast, H-LPAE_B4−S5−TMPTA_ exhibited a higher DNA-binding affinity of approximately 94%, which was much higher than that of the other H-LPAEs. Correspondingly, polyplexes formulated by the four H-LPAEs and DNA had sizes ranging from 162 to 470 nm. The polyplex formulated by H-LPAE_B4−S5−PET4A_ with DNA at a w/w ratio of 40:1 was 259 nm and 223 nm smaller than that of other w/w ratios (Fig. 3b). With increasing the w/w ratios, the particle size decreased, and DNA-binding affinity increased, which was mainly attributed to the increased DNA condensation and protection of H-LPAEs. Additionally, the zeta potential of all H-LPAE/DNA polyplexes exceed + 10.5 mV. With increasing the w/w ratios, almost zeta potential of H-LPAE_B4−S5−TMPTA/DNA_ polyplexes is gradually increasing, and more positive than that of other w/w ratios, which are + 8.5 mV and + 1.0 mV respectively (Fig. 3c). Furthermore, the morphology of the polyplexes was examined using TEM to assess its impact on cellular uptake. As illustrated in Fig. 3d and S10-S14, all four H-LPAE/DNA polyplexes exhibited a homogeneous and compact toroidal or spherical morphology, with diameters of approximately 250 nm, consistent with the DLS results. Taken together, these data indicated that the small size, positive zeta potential, and compact spherical morphology would favor cellular uptake and endosomal escape, thus facilitating efficient transfection.

Cellular uptake and endosomal escape of polyplexes posed significant challenges in gene delivery. To observe the intracellular distribution of the polyplexes, DNA was labeled with AF-647 (red fluorescence), and the cellular uptake of polyplexes and their intracellular distribution were first observed using fluorescence microscopy. Serum-free medium containing H-LPAE_B4−S5−PET4A_/ AF-647-labeled DNA polyplexes was added to 0.5 × 10^4^ cells and incubated for 4 h. The supernatant was removed and then washed three times with PBS. Finally, Hoechst33342 was added to the cells and incubated for 20 min, and then the cells were washed three times. As illustrated in Fig. 3e and S15, HeLa cells incubated with the H-LPAE_B4−S5−PET4A_/AF-647 DNA polyplexes exhibited intense red fluorescence, and the fluorescence mostly aggregated around the nucleus. These results suggested that the H-LPAE/DNA polyplexes showed high efficiency of cellular uptake, which may be attributed to their strong interaction with the cell membrane, multiple terminal groups and high zeta potential. The endosomal escape of polyplexes is a challenging barrier during gene delivery due to the low acidity of lysosomes, which promotes DNA degradation. Fluorescence microscopy was used to observe the distribution and co-localization of the two types of fluorescence. As shown in Fig. 3f and S15, most of the red fluorescence and green fluorescence did not overlap after incubation for 6 h. Additionally, the fluorescence signals from the Pearson’s correlation coefficient (PCC) curves were noticeably inconsistent or detached, indicating the superior endosomal escape of H-LPAE_B4−S5−PET4A_ (Fig. 3f). All results demonstrated that the linear oligomer combination sequential branching strategy endows H-LPAE with a higher charge density to promote cellular uptake and endosomal escape of the polyplexes.

### Optimized H-LPAEs exhibit superior gene transfection activity

**Fig. 4:**
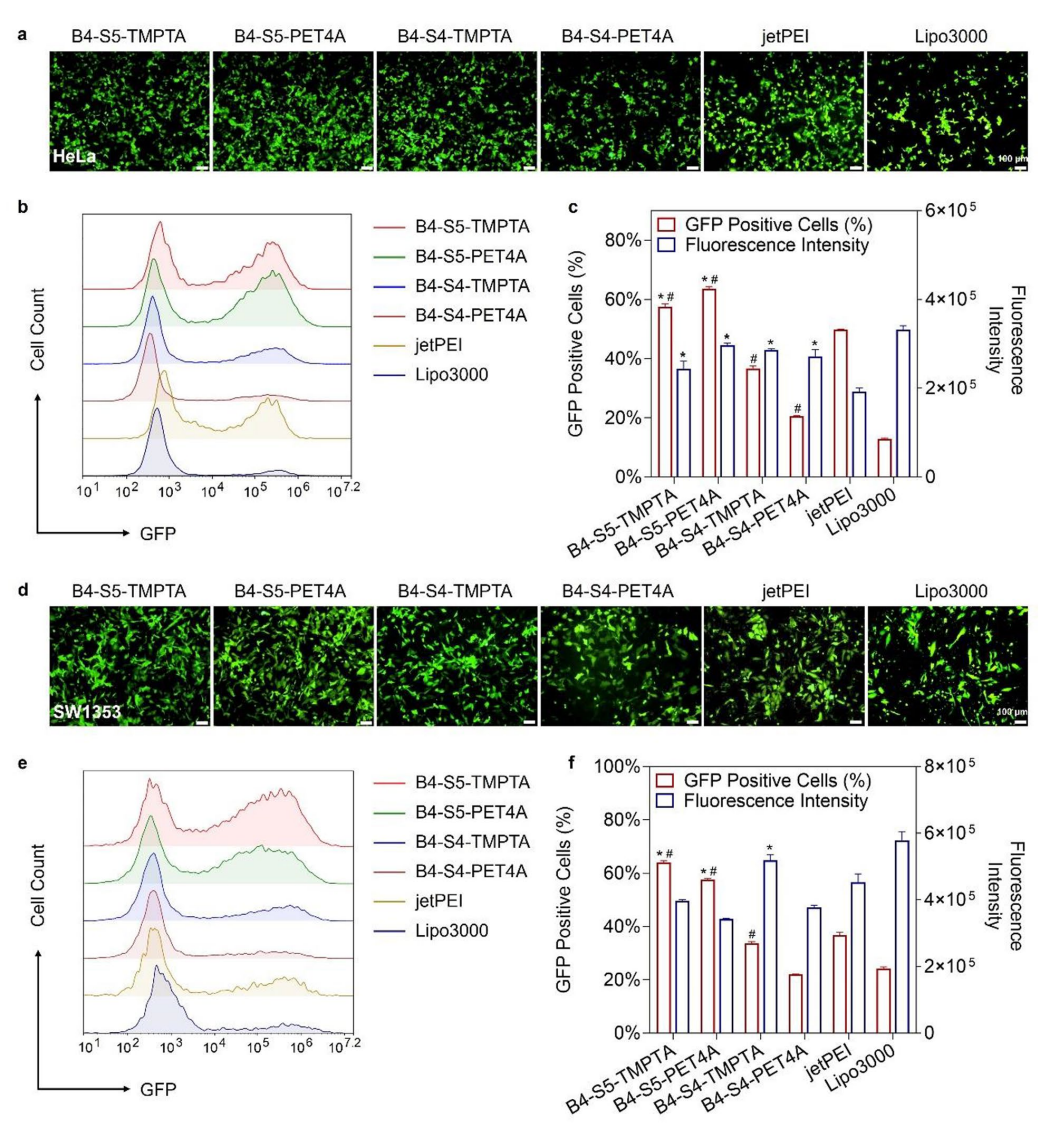
Optimized H-LPAE/DNA polyplexes show great superiority in gene transfection for various cells. **(a)** Representative fluorescence images of HeLa cells after transfection with various H-LPAEs/DNA polyplexes. Scale bars, 100 μm; **(b)** Histogram distribution of GFP-positive populations in HeLa cells after transfection with various H-LPAEs/DNA polyplexes; **(c)** The percentage of GFP-positive cell and mean fluorescence intensity of HeLa cells after transfection with various H-LPAEs/DNA polyplexes, compared to jetPEI (*, *p* < 0.05) and Lipo3000 (^#^, *p* < 0.05) (n = 3, Student’s t test); **(d)** Representative fluorescence images of SW1353 cells after transfection with various H-LPAEs/DNA polyplexes. Scale bars, 100 μm; **(e)** Histogram distribution of GFP-positive populations in SW1353 cells after transfection with various H-LPAEs/DNA polyplexes; **(f)** The percentage of GFP-positive cell and mean fluorescence intensity of SW1353 cells after transfection with various H-LPAEs/DNA polyplexes. For the percentage of GFP-positive cell, compared to jetPEI (*, *p* < 0.05), and Lipo3000 (^#^, *p* < 0.05); For the mean fluorescence intensity, compared to jetPEI (*, *p* < 0.05), and Lipo3000 (^#^, *p* < 0.05). Data is represented as mean ± SD (n = 3, Student’s t test)

The w/w ratio of polymer to DNA is a crucial factor that significantly influences DNA delivery both in vitro and in vivo. Therefore, we quantitatively and qualitatively evaluated the transfection efficiency of polyplexes at w/w ratios of 20:1, 40:1, and 60:1. As positive controls, the commercial transfection reagents jetPEI and Lipo3000 were used. After 4-hour incubation, the polyplex-containing medium without FBS was replaced with culture medium containing 10% FBS, and the cells were further cultured for 44 h. As shown in Fig. 4a, at a w/w ratio of 20:1, all four H-LPAE/DNA polyplexes exhibited much higher gene transfection efficiency and stronger green fluorescence in HeLa Cells. Moreover, H-LPAE_B4−S5−PET4A_/DNA polyplexes demonstrated higher gene transfection efficiency than other polyplexes, with a high percentage of GFP-positive cells (64.4%) and a mean fluorescence intensity of 302,140 RLU (Fig. 4b and **c**). In comparison, relatively weak green fluorescence was observed in the cells after transfection with four H-LPAEs at a w/w ratio of 60:1 (Figure S17), which may be due to the fact that a high polymer dosage increases cytotoxicity after transfection. Efficient DNA delivery from multiple tissues to multiple cells is a crucial factor in evaluating the broad applicability of H-LPAEs for gene therapy. Therefore, in addition to HeLa cells, we selected various cell lines derived from different tissues, including SW1353 cells, rat Schwann cell 96 (RSC96), and difficult-to-transfect bone marrow-derived stem cells (BMSCs), to assess the DNA transfection efficiency of H-LPAEs. As shown in Fig. 4d and f and Figure S18-S19, all four H-LPAEs exhibited high transfection efficiency at three different w/w ratios, especially at a w/w ratio of 20:1. Notably, H-LPAE_B4−S5−PET4A_ achieved significantly higher GFP transfection efficiency (58.0%) compared to jetPEI (35.5%) and Lipo3000 (23.3%) in SW1353 cells. Even in difficult-to-transfect stem cells, H-LPAE_B4−S5−TMPTA_ demonstrated remarkably high transfection efficiency. Flow cytometry analysis further confirmed these findings, showing its transfection efficiencies exceeding 33.4% at w/w ratios of 20:1, which were 1.6-fold higher than jetPEI and 2.0-fold higher than Lipo3000 (Figure S20-21).

**Fig. 5:**
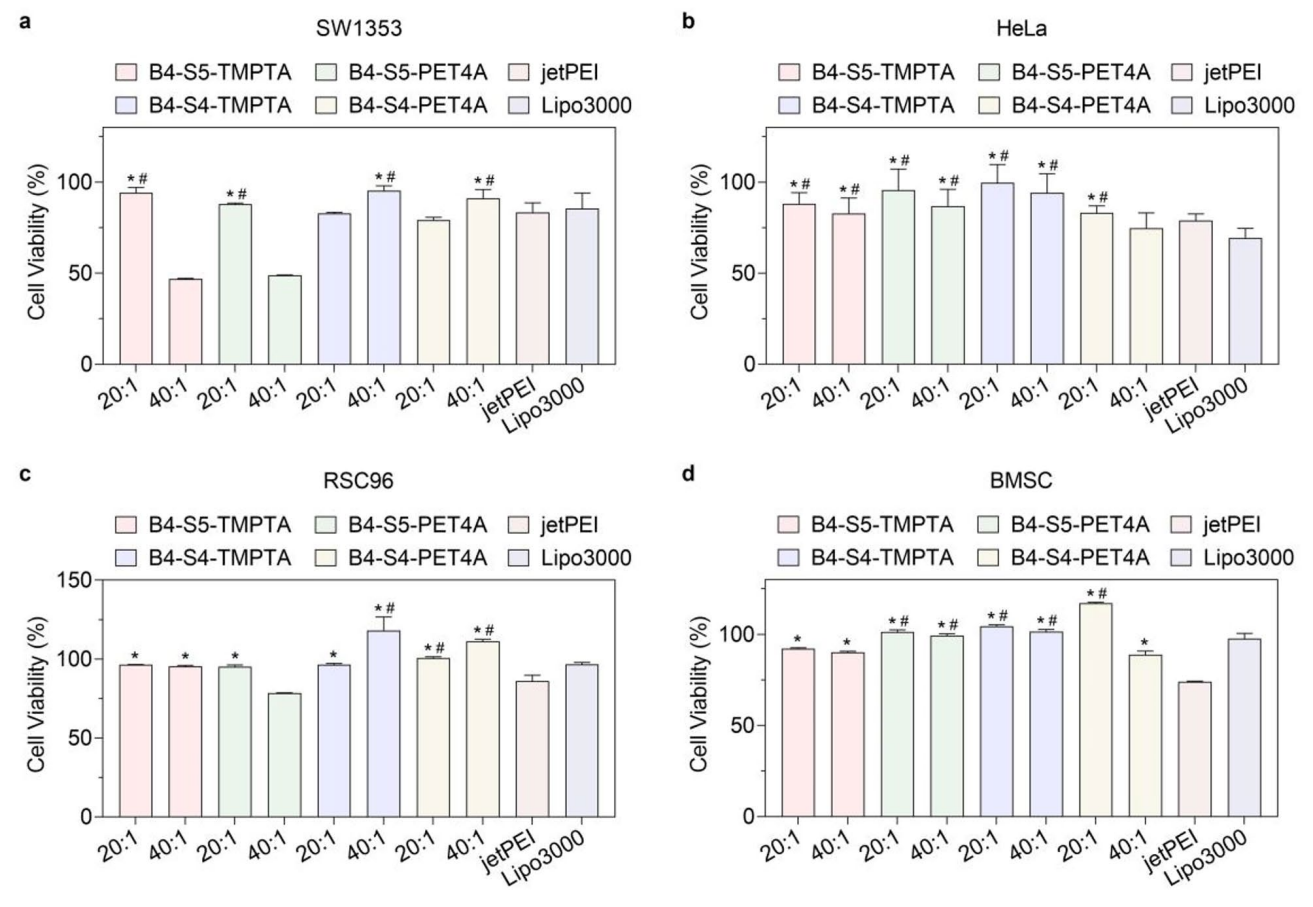
All H-LPAEs show high cell viability for various cells. (a-d) Cell viability of **(a)** SW1353 cells, **(b)** HeLa cells, **(c)** RSC96 cell, and **(d)** BMSC cells after transfection with various H-LPAE/DNA polyplexes at the w/w ratios of 20 :1 and 40:1, compared to jetPEI (*, *p* < 0.05) and Lipo3000 (^#^, *p* < 0.05). Data is represented as mean ± SD (n = 3, Student’s t test)

In addition to transfection efficiency, the cytotoxicity of HPAEs after transfection was determined for clinical translation. AlamarBlue results revealed that the cell viability of almost all H-LPAEs exceeded 88% at w/w ratios of 20:1 in HeLa cells, SW1353 cells, RSC96 cells, and BMSC cells (Fig. 5a and d), which is higher than the activity of jetPEI and Lipo3000. In particular, H-LPAE_B4−S5−PET4A_ maintained high cell viability (> 95%) in HeLa cells, which was 1.2-fold higher than that of jetPEI and 1.4-fold higher than that of Lipo3000 (Fig. 5b). As the w/w ratio increased, the H-LPAE_B4−S5−TMPTA_/DNA polyplexes did not induce obvious cytotoxicity. Even in BMSC cells, H-LPAE_B4−S5−TMPTA_ maintained higher cell viability (> 97%) than jetPEI and Lipo3000 (Fig. 5d). Previous reports also confirmed that HPAEs exhibit low cytotoxicity, mainly attributed to their hydrophilic, biodegradable backbone structure [20]. Taken together, these results demonstrated that H-LPAEs have a high gene transfection efficiency without causing noticeable cytotoxicity in various cells, especially at a relatively low w/w ratio, which further provides an important platform for *TRAIL* gene delivery to cancer cells.

### Optimized H-LPAEs efficiently mediates *TRAIL* gene transfection to induce apoptosis in cancer cells

**Fig. 6:**
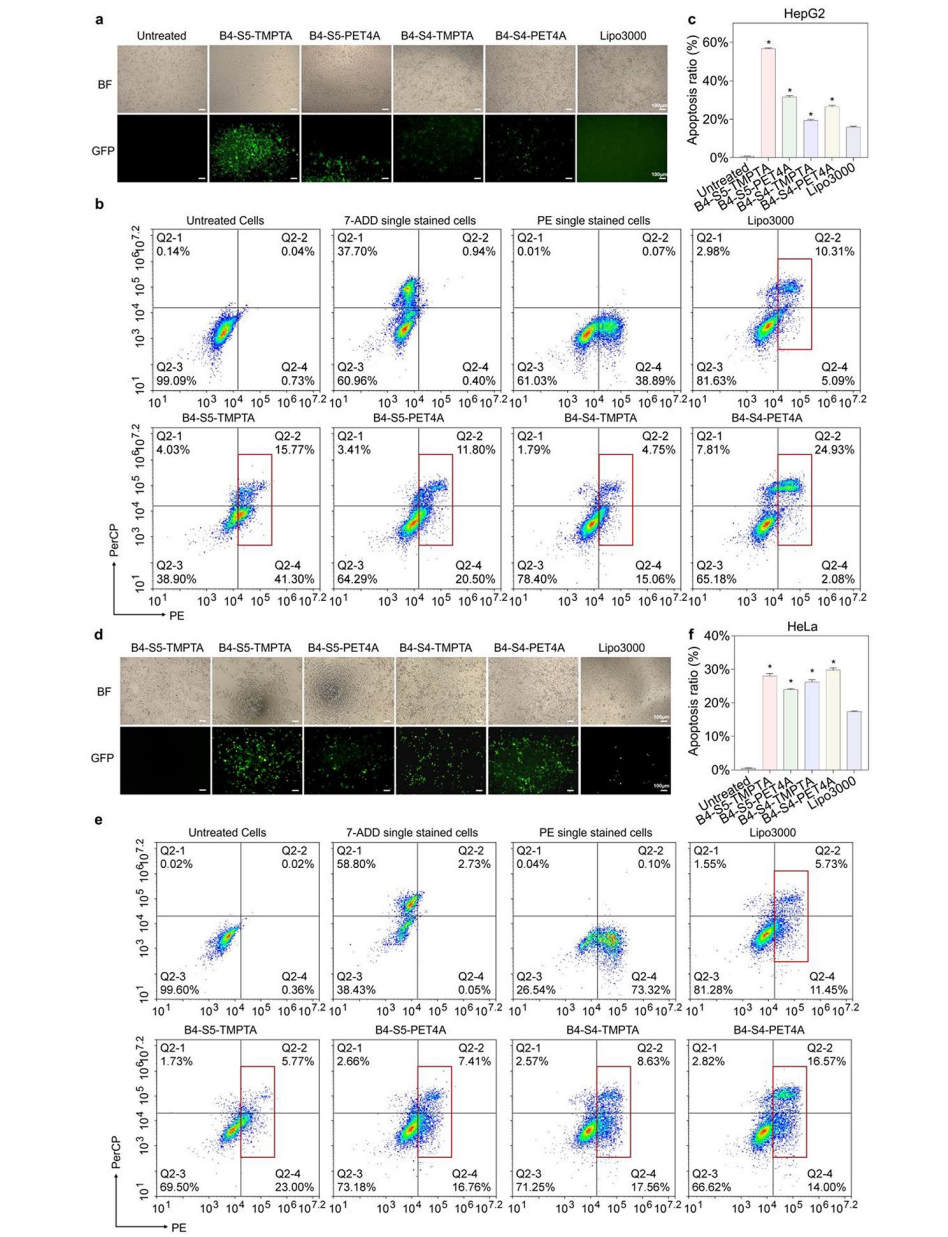
Optimized H-LPAEs showed high *TRAIL* gene transfection efficiency to induce apoptosis in various cancer cells. **(a)** Representative microscopy images and fluorescence images of HepG2 cells after transfection with various H-LPAEs/DNA polyplexes. Scale bars, 100 μm; **(b)** Flow cytometry analysis of apoptosis efficiency in HepG2 cells after transfection with various H-LPAEs/DNA polyplexes; **(c)** Quantification of the apoptosis efficiency corresponding to the (b) (*p < 0.05 compared to Lipo3000, n = 3); **(d)** Representative microscopy images and fluorescence images of HeLa cells after transfection with various H-LPAEs/DNA polyplexes. Scale bars, 100 μm; **(e)** Flow cytometry analysis of apoptosis efficiency in HeLa cells after transfection with various H-LPAEs/DNA polyplexes; **(f)** Quantification of the apoptosis efficiency corresponding to the **(e)**. Data is represented as mean ± SD, *p < 0.05 compared to Lipo3000 (n = 3). BF represents bright field

Numerous studies have demonstrated that intracellular transfection of *TRAIL* gene can achieve efficient apoptosis of cancer cells, making it a promising approach for cancer therapy [21, 22]. In addition, many studies have focused on specific cancer cells or cancer [23]. Therefore, the development of a technology platform for gene vectors applicable to various cancer cells is necessary for cancer therapy. Preliminary results indicated that H-LPAE_B4−S5−TMPTA_ and H-LPAE_B4−S5−PET4A_ could highly efficiently mediate GFP transfection in SW1353 cells and HeLa cells, while maintaining high cellular activity. This suggested that the optimized H-LPAE is suitable for transfection in different cancer cell types. As shown in Figure S23-S24, H-LPAE_B4−S5−TMPTA_ induced apoptosis in SW1535 cells by more than 29% after TRAIL-mediated transfection for 48 h at a w/w ratio of 20:1, which was higher than that of Lipo3000. The significant GFP expression further demonstrated the ability of H-LPAE_B4−S5−TMPTA_ to efficiently induce apoptosis in SW1353 cells (Figure S22). Furthermore, we evaluated the apoptosis efficacy in hepatocellular carcinoma cells and the liver cancer therapeutic effect of H-LPAE_B4−S5−TMPTA_. As shown in Fig. 6a-c, H-LPAE_B4−S5−TMPTA_ effectively mediated TRAIL gene-induced apoptosis with an efficiency of 56.7% in HepG2 cells, which was 3.6-fold higher than that of Lipo3000. Importantly, apoptotic cells exhibited lower cell viability, poorer cell morphology, and higher GFP intensity, indicating that the optimized H-LPAE showed great potential for inducing cancer cell apoptosis and the treatment of hepatocellular carcinoma. Finally, the optimized H-LPAE/TRAIL DNA polyplexes efficiently mediated HeLa cell apoptosis, with an apoptosis efficiency of 28.1% (Fig. 6d-f). Taken together, these results suggest that H-LPAE HPAEs are efficient vectors for *TRAIL* DNA delivery, showing their great potential in cancer therapy and may advance our understanding of cancer gene therapy.

### Locally administered H-LPAE_B4−S5−TMPTA_ enable TRAIL DNA delivery to HepG2 xenograft tumors and slow Tumor growth

To further evaluate the in vivo anticancer efficacy, we randomly divided the subcutaneous xenograft HepG2 tumors into three groups: control empty H-LPAE_B4−S5−TMPTA_, empty H-LPAE_B4−S5−TMPTA_ with IV vorinostat, or TRAIL H-LPAE_B4−S5−TMPTA_ with IV vorinostat. Vorinostat was chosen for in vivo testing due to its promising in vitro anti-cancer activity when combined with TRAIL [24]. Moreover, this drug has already been approved for clinical use in humans to treat cutaneous T cell lymphoma [25].

**Fig. 7:**
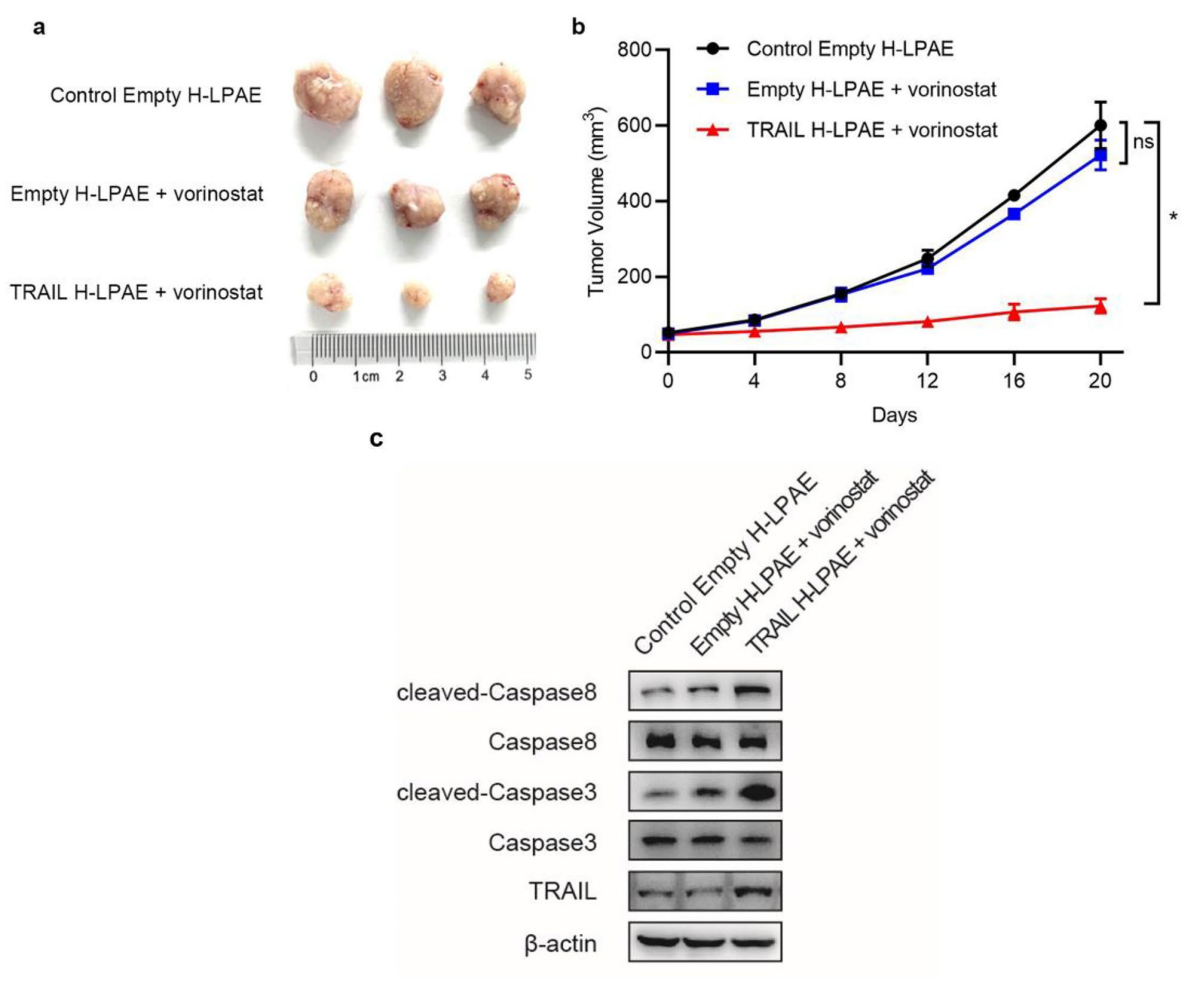
H-LPAE_B4−S5−TMPTA_ administered intratumorally with systemic vorinostat slow the growth of HepG2 subcutaneous xenografts. **(a)** The xenograft tumor model was established via subcutaneous inoculation of HepG2 cells in BALB/c-nude mice. Mice were randomly divided into three groups and received intratumoral injections of H-LPAE_B4−S5−TMPTA_ synthesized with empty plasmid (Control Empty H-LPAE), intratumoral injections of H-LPAE_B4−S5−TMPTA_ synthesized with empty plasmid and intravenous administration of vorinostat (Empty H-LPAE + vorinostat), and intratumoral injections of H-LPAE_B4−S5−TMPTA_ synthesized with TRAIL plasmid and intravenous administration of vorinostat (TRAIL H-LPAE + vorinostat). The tumors were excised on day 20 and three representative mice in each group were shown. n = 3. **(b)** The tumor volume progression of nude mice within the observation period. Data is represented as mean ± SD; ns, not significant; *p < 0.05; Student’s t-test. n = 3. **(c)** Western blot analysis of cleaved Caspase 8, Caspase 8, cleaved Caspase 3, Caspase 3, TRAIL, β-actin in tumor tissues between different groups

To control for potential immunogenicity or toxicity from expression of a foreign protein and isolate the TRAIL-mediated anti-tumor effect, H-LPAE_B4−S5−TMPTA_ harboring empty plasmid were selected. When the tumor volume reached approximately 50 mm^3^, every 4 days animals received intratumoral injection of TRAIL H-LPAE_B4−S5−TMPTA_ at a 10 µg DNA dose and/or intravenous administration of vorinostat at an estimated blood concentration of 10 µM. As shown in Fig. 7a and **b**, the tumor growth curve indicated that no anticancer effect was observed from vorinostat alone (*p* > 0.05). However, TRAIL H-LPAE_B4−S5−TMPTA_ with vorinostat dramatically slowed tumor growth in comparison to animals receiving control empty H-LPAE_B4−S5−TMPTA_ (*p* < 0.05). In addition, western blot showed that TRAIL H-LPAE_B4−S5−TMPTA_ with vorinostat induced activation of caspase-8 and − 3 in tumor tissues, suggesting successful induction of apoptotic pathway. Meanwhile, western blot showed that TRAIL protein increased after intratumoral injection of TRAIL H-LPAE_B4−S5−TMPTA_, indicting successful delivery of *TRAIL* DNA (Fig. 7c).

## Conclusions

Duo to the challenge of achieving safe and effective *TRAIL* gene delivery of the *TRAIL* gene in cancer gene therapy, this study introduced a novel approach called the linear oligomer combination branched strategy to synthesize a new structure H-LPAEs with MW ranging from 11.8 kg/mol to 19.0 kg/mol. The oligomer combination branched strategy enhances the uniform distribution of the linear segments and branching units of H-LPAEs, their DNA condensation ability and resulting in positively charged H-LPAE/DNA polyplexes. In various type of cell lines, H-LPAEs mediated high levels of gene transfection efficiency, particularly in HeLa and SW1353 cells, exceeded 64.4% and 64.3%, respectively, while maintaining superior the safety profile. Importantly, the tumor cell apoptosis results demonstrated that the optimized H-LPAEs high-efficiently mediated *TRAIL* DNA transfection, achieving transfection efficiencies of up to 58% in HepG2 cells, inducing tumor cell apoptosis in vitro, and showing anticancer efficacy in vivo. These findings highlight a highly efficient gene vector candidate for *TRAIL* DNA delivery, and may advance our understanding of cancer gene therapy.

### Electronic supplementary material

Below is the link to the electronic supplementary material.

Supplementary Material 1

## Data Availability

All data used to generate these results are available in the main text and supporting information.
