# Supplementary material for: Superior TRAIL gene expression and cancer cell apoptosis mediated by highly branched-linear poly(β-amino ester)s
Source: J Nanobiotechnology. 2023 Oct 28;21:394. doi: 10.1186/s12951-023-02169-7 (PMC10612241; doi:10.1186/s12951-023-02169-7)
Supplement: Supplementary file 1 — Supplementary Material 1 [file 12951_2023_2169_MOESM1_ESM.docx]

**Supplementary Information**

**Superior TRAIL Gene Expression and Cancer Cell Apoptosis Mediated by Highly Branched-Linear Poly(β-amino ester)s**

Yitong Zhao^a,#^, Tao Bo^b,#^, Chenfei Wang^c,#,^*, Dingjin Yao^b,c^ , Chaolan Pan^c^, Weiyi Xu^c^, Hao Zhou^d^, Ming Li^c,^*, Si Zhang ^a,b,^*

*^a^*School of Medicine, Anhui University of Science and Technology, Huainan, Anhui, 232000, China.

*^b^NHC Key Laboratory of Glycoconjugate Research, Department of Biochemistry and Molecular Biology, School of Basic Medical Sciences, Fudan University, Shanghai, 200032, China.*

*^c^Department of Dermatology, Children's Hospital of Fudan University, National Children's Medical Center, 399 Wanyuan Road, Shanghai 201102, China*

*^d^State Key Laboratory of Medicinal Chemical Biology, Tianjin Key Laboratory of Protein Science, and College of Life Sciences, Nankai University, Tianjin 300071, China*

*Email: [zhangsi@fudan.edu.cn](mailto:zhangsi@fudan.edu.cn); [mingli@fudan.edu.cn](mailto:mingli@fudan.edu.cn); wangchenfei@fudan.edu.cn

^#^These authors contributed equally: Yitong Zhao; Tao Bo; and Chenfei Wang

**Table S1.** Feed ratio of monomers for the synthesis of LPAE_B4-S4_ polymer

|  | Feed Ratio  (mole) | Mass Ratio  (g) |
| --- | --- | --- |
| S4 | 2.4 | 0.178 |
| B4 | 2 | 0.476 |
| E7 | 0.6 | 0.094 |

**Table S2.** Feed ratio of monomers for the synthesis of LPAE_B4-S5_ polymer

|  | Feed Ratio  (mole) | Mass Ratio  (g) |
| --- | --- | --- |
| S5 | 2.4 | 0.206 |
| B4 | 2 | 0.476 |
| 122 | 0.6 | 0.115 |

**Table S3.** Feed ratio of monomers for the synthesis of HPAE_B4-S4-TMPTA_ polymer

|  | Feed Ratio  (mole) | Mass Ratio  (g) |
| --- | --- | --- |
| LPAE_B4-S4_ |  | 0.654 |
| TMPTA | 0.13 | 0.039 |
| E7 | 0.6 | 0.094 |

**Table S4.** Feed ratio of monomers for the synthesis of HPAE_B4-S4-PET4A_ polymer

|  | Feed Ratio  (mole) | Mass Ratio  (g) |
| --- | --- | --- |
| LPAE_B4-S4_ |  | 0.654 |
| PET4A | 0.13 | 0.039 |
| E7 | 0.6 | 0.094 |

**Table S5.** Feed ratio of monomers for the synthesis of HPAE_B4-S5-TMPTA_ polymer

|  | Feed Ratio  (mole) | Mass Ratio  (g) |
| --- | --- | --- |
| LPAE_B4-S5_ |  | 0.682 |
| TMPTA | 0.13 | 0.039 |
| 122 | 0.6 | 0.115 |

**Table S6.** Feed ratio of monomers for the synthesis of HPAE_B4-S5-PET4A_ polymer

|  | Feed Ratio  (mole) | Mass Ratio  (g) |
| --- | --- | --- |
| LPAE_B4-S5_ |  | 0.682 |
| PET4A | 0.1 | 0.035 |
| 122 | 0.6 | 0.115 |

**Table S7.** MW and *Đ* of O-LPAE_B4-S4_^1^ during polymerization

| Time (h) | *M*_n_ (kDa) | *M*_w_ (kDa) | *Đ* |
| --- | --- | --- | --- |
| 0 | 0.9 | 1.1 | 1.2 |
| 2.5 | 2.2 | 3.1 | 1.4 |
| 4.5 | 2.6 | 4.0 | 1.6 |
| 6 | 2.8 | 4.5 | 1.6 |

**Table S8.** MW and *Đ* of HPAE_B4-S4-TMPTA_^1^ during polymerization

| Time (h) | *M*_n_ (kDa) | *M*_w_ (kDa) | *Đ* |
| --- | --- | --- | --- |
| 0 | 3.4 | 5.3 | 1.5 |
| 0.5 | 3.8 | 5.7 | 1.5 |
| 5 | 5.7 | 10.2 | 1.8 |
| 6.5 | 5.6 | 10.8 | 1.9 |
| End capping | 6.0 | 11.8 | 2.0 |

**Table S9.** MW and *Đ* of O-LPAE_B4-S4_^2^ during polymerization

| Time (h) | *M*_n_ (kDa) | *M*_w_ (kDa) | *Đ* |
| --- | --- | --- | --- |
| 0 | 0.9 | 1.1 | 1.2 |
| 6 | 2.6 | 4.2 | 1.6 |

**Table S10.** MW and *Đ* of HPAE_B4-S4-PET4A_^2^ during polymerization

| Time (h) | *M*_n_ (kDa) | *M*_w_ (kDa) | *Đ* |
| --- | --- | --- | --- |
| 1 | 3.6 | 5.8 | 1.6 |
| 3 | 4.9 | 9.0 | 1.8 |
| 5 | 5.4 | 10.9 | 2.0 |
| End capping | 5.7 | 11.9 | 2.1 |

**Table S11.** MW and *Đ* of O-LPAE_B4-S5_^1^ during polymerization

| Time (h) | *M*_n_ (kDa) | *M*_w_ (kDa) | *Đ* |
| --- | --- | --- | --- |
| 0 | 1.0 | 1.2 | 1.2 |
| 1.5 | 1.1 | 1.5 | 1.3 |
| 6.5 | 2.3 | 3.4 | 1.5 |
| 12.5 | 2.8 | 4.5 | 1.6 |

**Table S12.** MW and *Đ* of H-LPAE_B4-S5-TMPTA_^1^ during polymerization

| Time (h) | *M*_n_ (kDa) | *M*_w_ (kDa) | *Đ* |
| --- | --- | --- | --- |
| 0 | 5.6 | 12.0 | 2.1 |
| 1.5 | 6.3 | 14.4 | 2.3 |
| 2 | 5.9 | 15.7 | 2.7 |
| End capping | 6.4 | 15.4 | 2.4 |

**Table S13.** MW and *Đ* of O-LPAE_B4-S5_^2^ during polymerization

| Time (h) | *M*_n_ (kDa) | *M*_w_ (kDa) | *Đ* |
| --- | --- | --- | --- |
| 0 | 1.0 | 1.2 | 1.2 |
| 3 | 1.8 | 2.3 | 1.3 |
| 10 | 2.7 | 4.3 | 1.6 |
| 12.5 | 2.8 | 4.6 | 1.6 |

**Table S14.** MW and *Đ* of H-LPAE_B4-S5-PET4A_^2^ during polymerization

| Time (h) | *M*_n_ (kDa) | *M*_w_ (kDa) | *Đ* |
| --- | --- | --- | --- |
| 0 | 5.8 | 13.3 | 2.3 |
| 1.5 | 6.8 | 17.2 | 2.5 |
| 2.5 | 6.5 | 20.6 | 3.2 |
| End capping | 5.8 | 19.0 | 3.3 |

_
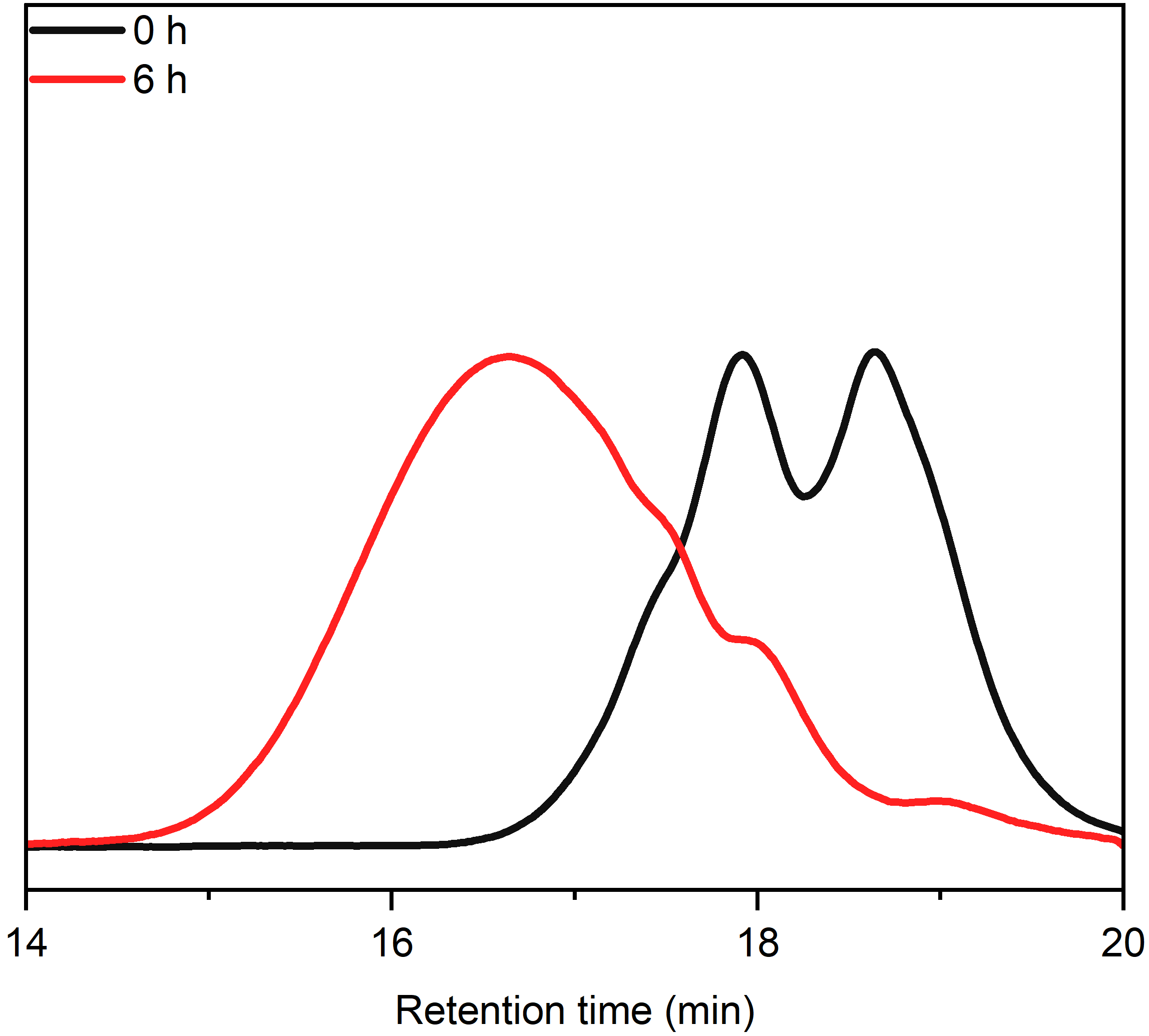
_

**Figure S1.** GPC traces of O-LPAE_B4-S4_^2^ during the polymerization process

_
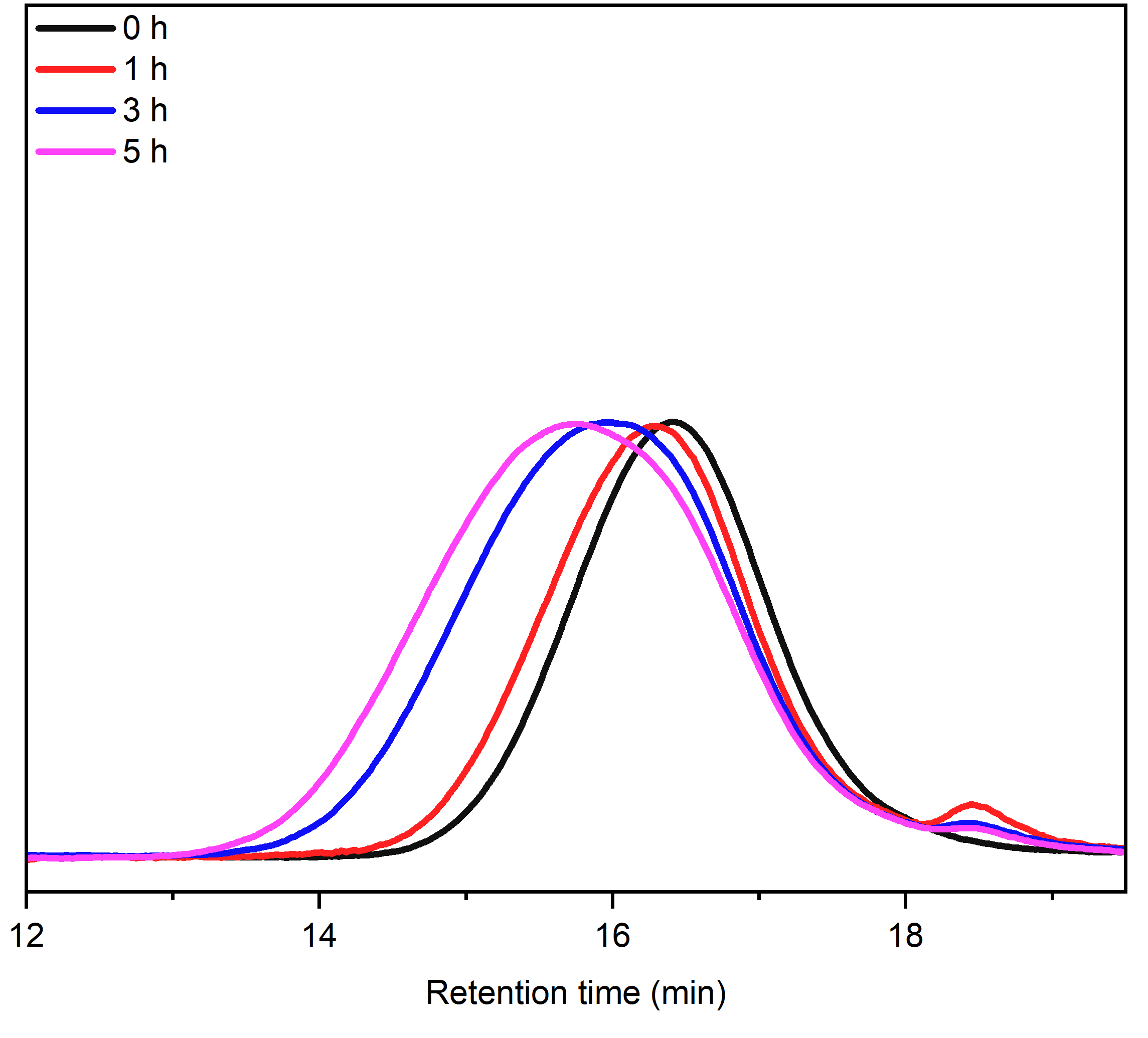
_

**Figure S2.** GPC traces of H-LPAE_B4-S4-PET4A_^2^ during the polymerization process


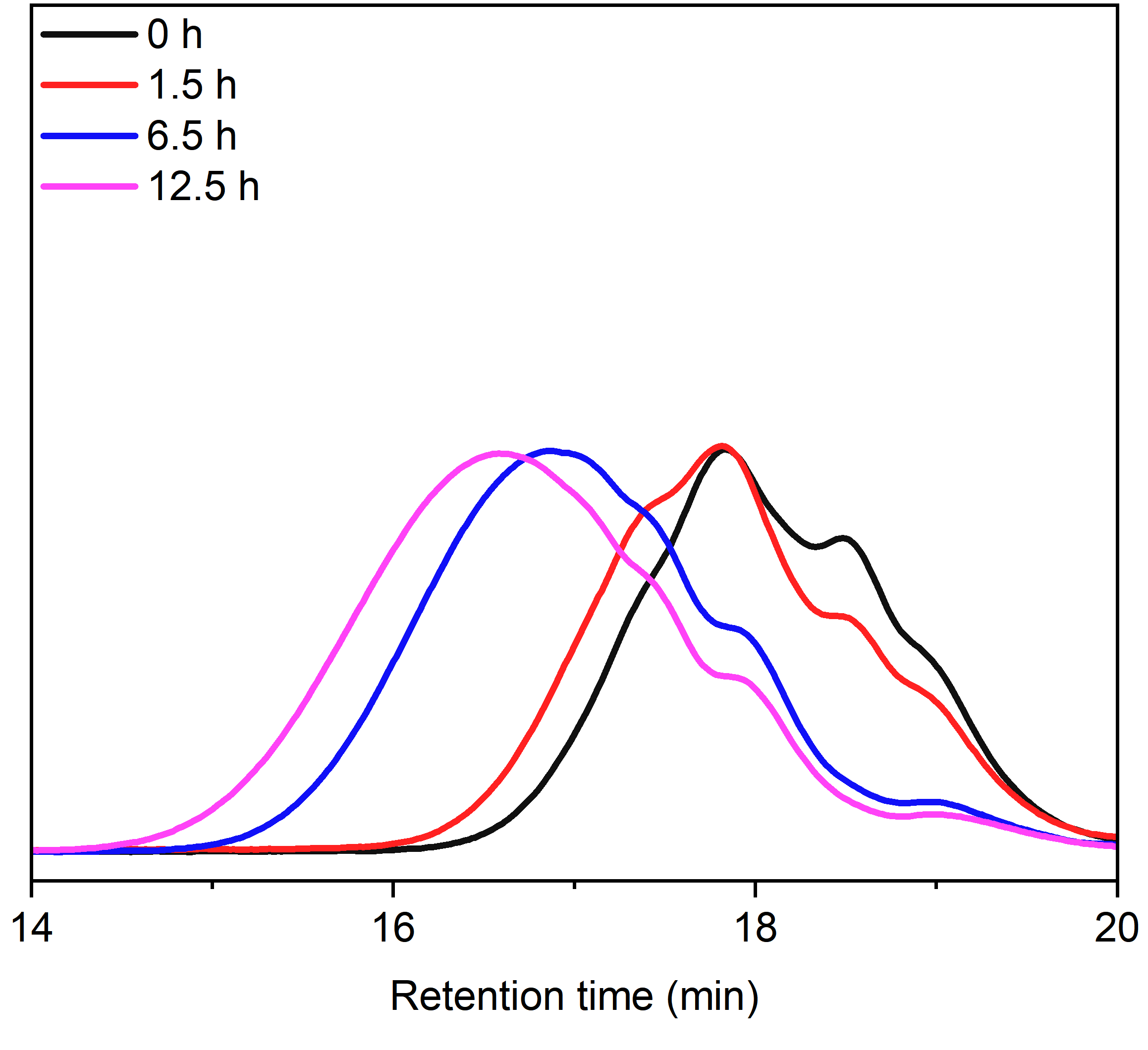


**Figure S3.** GPC traces of O-LPAE_B4-S5-ac_^1^ during the polymerization process


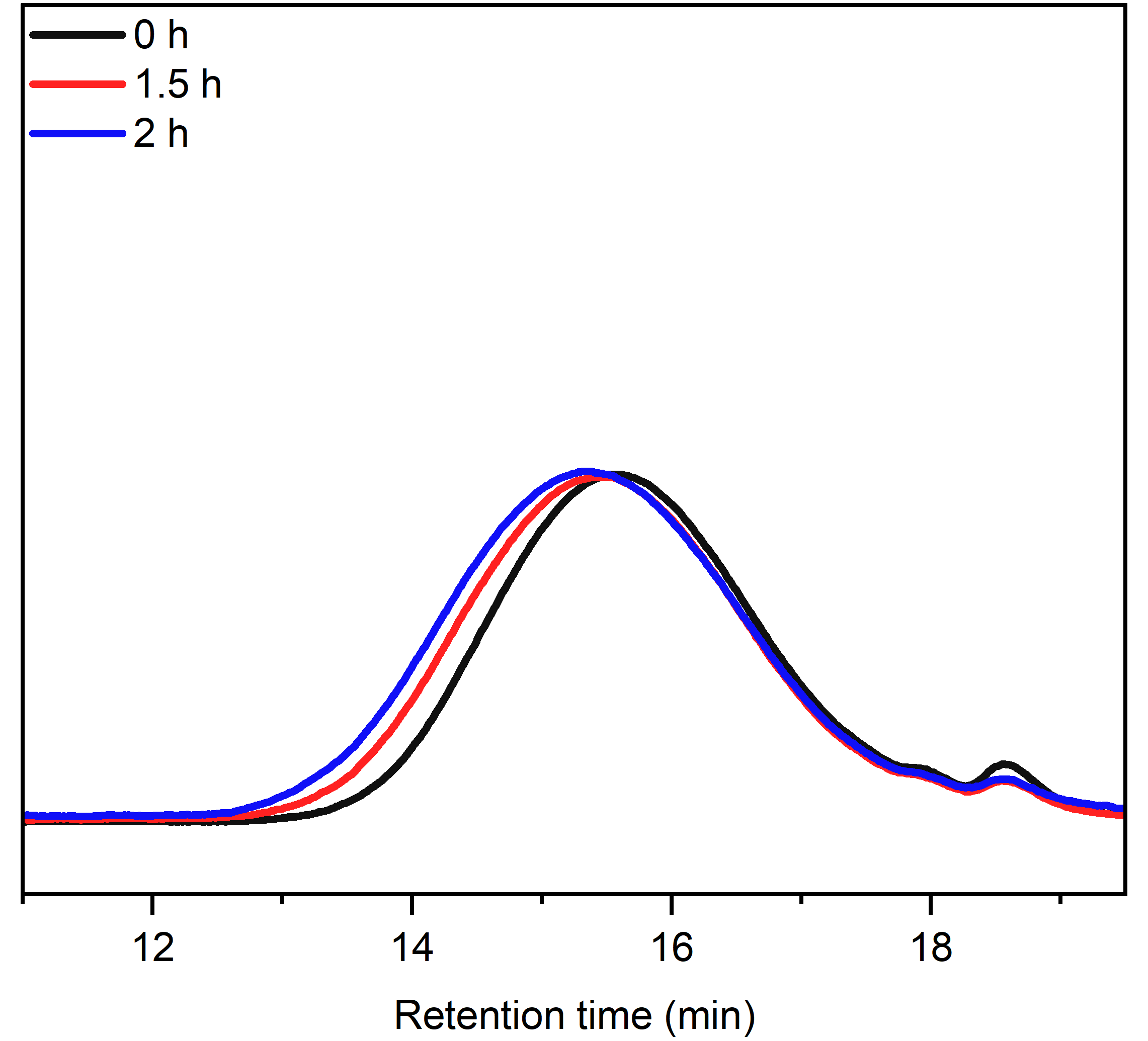


**Figure S4.** GPC traces of HPAE_B4-S5-TMPTA_^1^ during the polymerization process

_
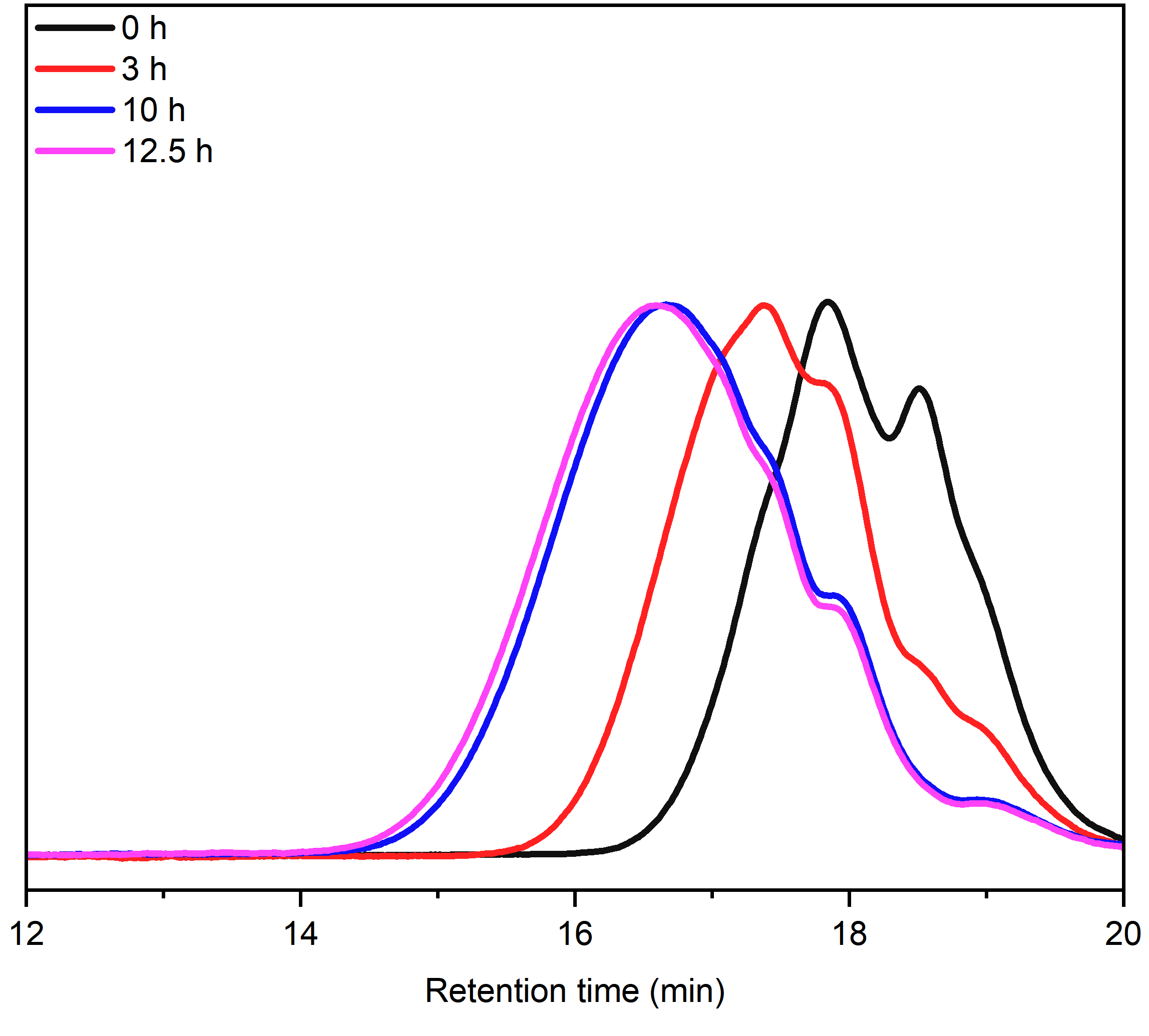
_

**Figure S5.** GPC traces of O-LPAE_B4-S5_^2^ during the polymerization process

_
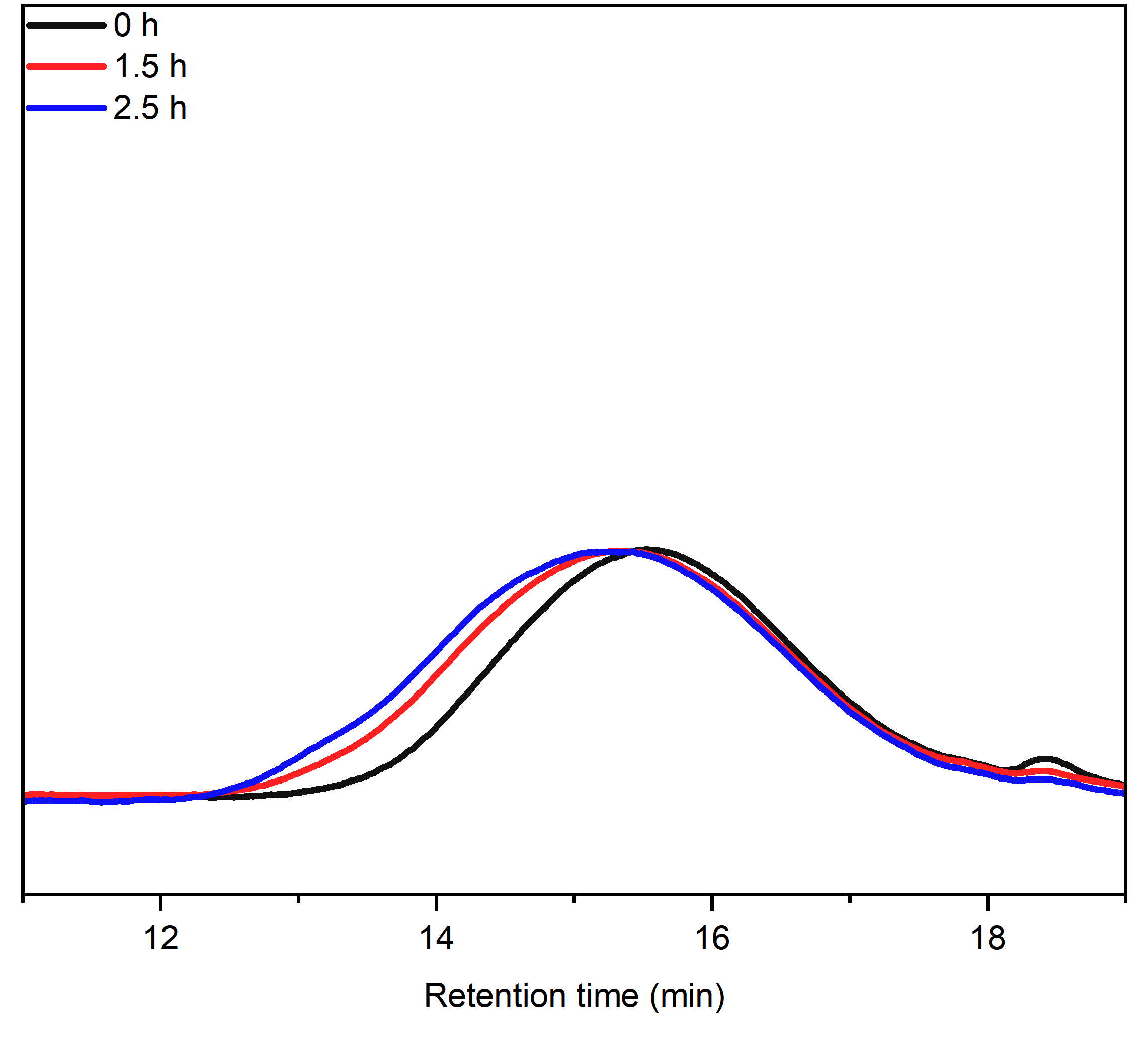
_

**Figure S6.** GPC traces of H-LPAE_B4-S5-PET4A_^2^ during the polymerization process

_
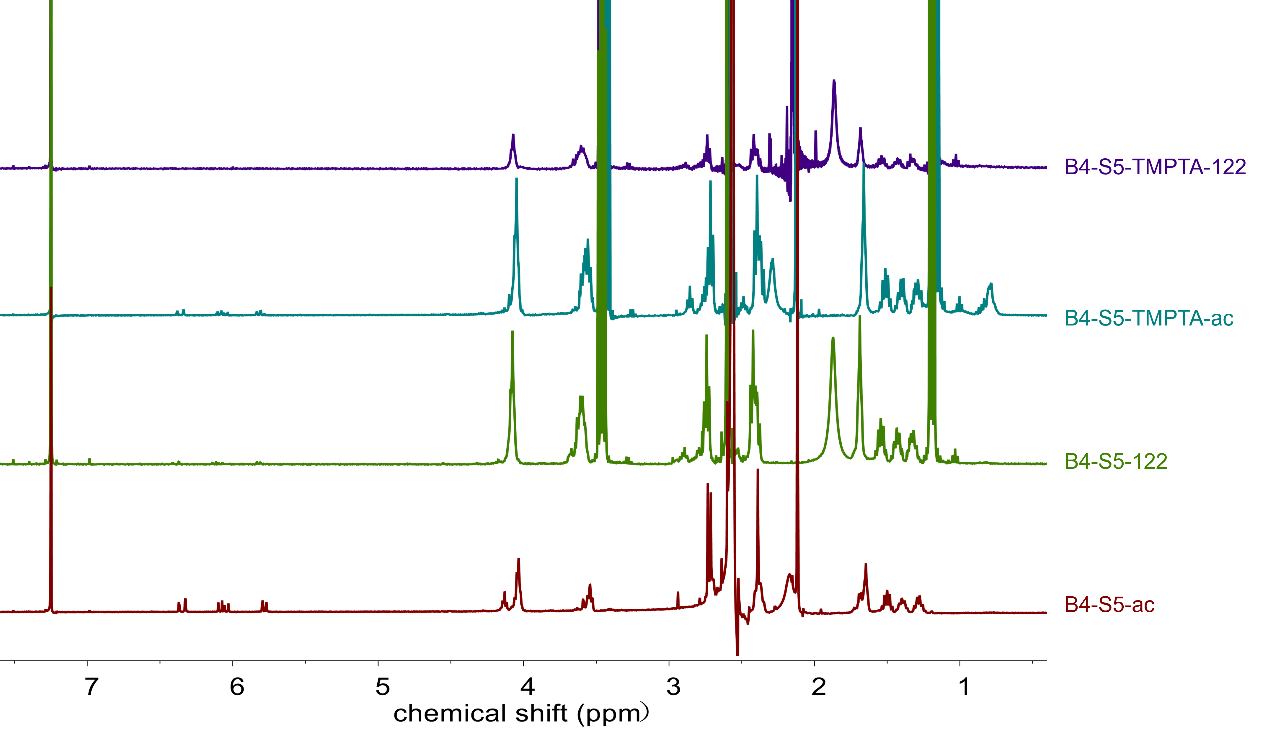
_

**Figure S7.** ^1^H NMR spectrum of O-LPAE_B4-S5_^1^ and H-LPAE_B4-S5-TMPTA_^1^ before and after end-capping

_
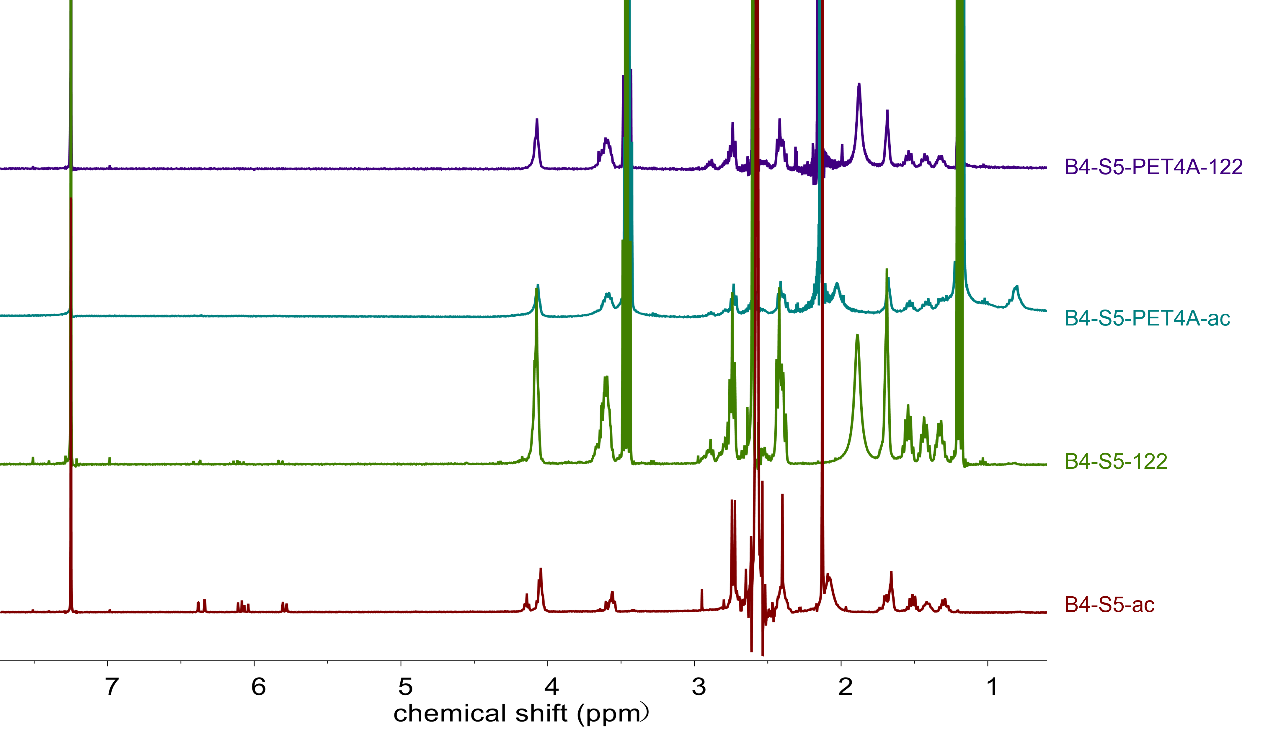
_

**Figure S8.** ^1^H NMR spectrum of O-LPAE_B4-S5_^2^ and H-LPAE_B4-S5-PET4A_^2^ before and after end-capping

_
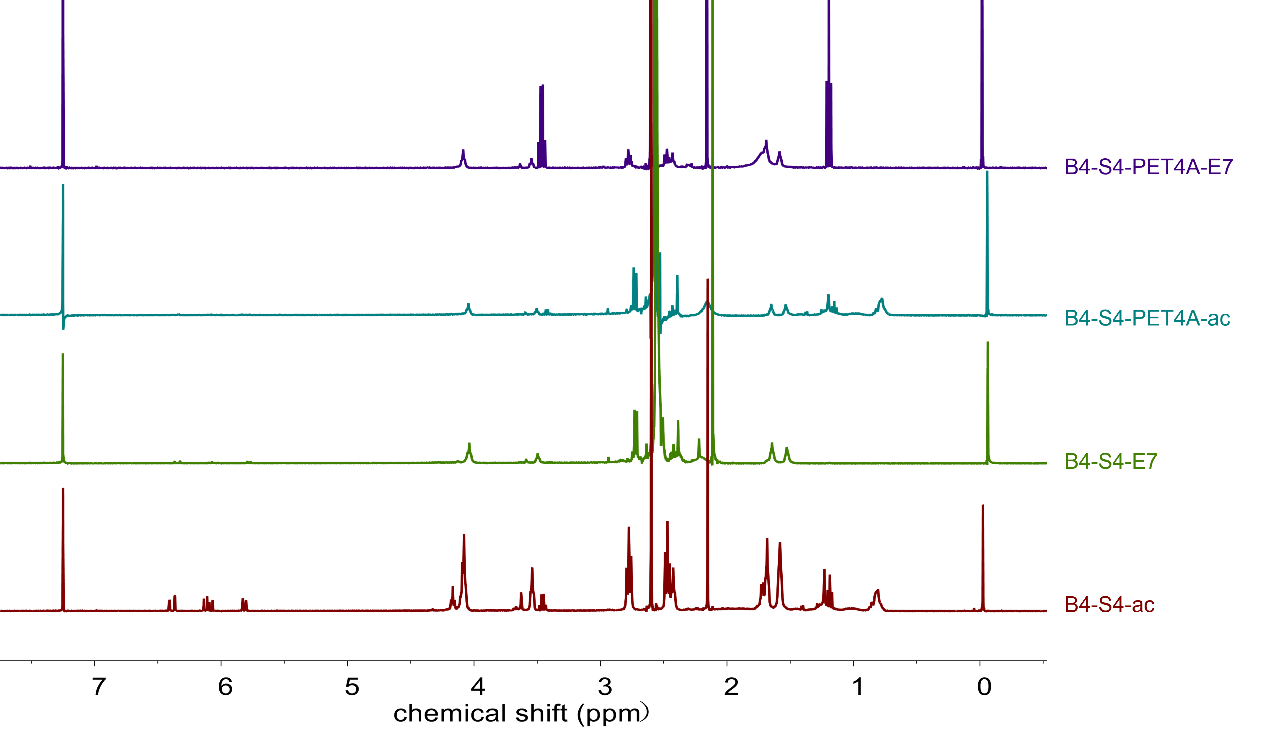
_

**Figure S9.** ^1^H NMR spectrum of O-LPAE_B4-S4_^2^ and H-LPAE_B4-S4-PET4A_^2^ before and after end-capping

_
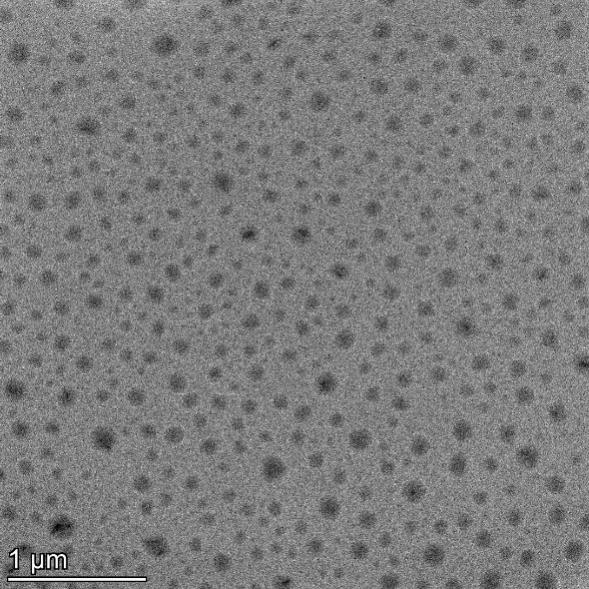
_

**Figure S10.** Representative TME images of H-LPAE_B4-S5-PET4A_/DNA polyplexes. Scale bars, 1 μm

_
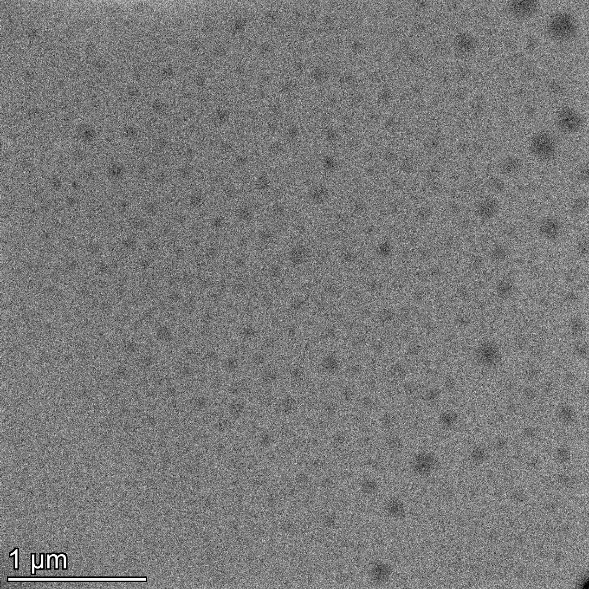
_

**Figure S11.** Representative TME images of H-LPAE_B4-S4-TMPTA_/DNA polyplexes. Scale bars, 1 μm


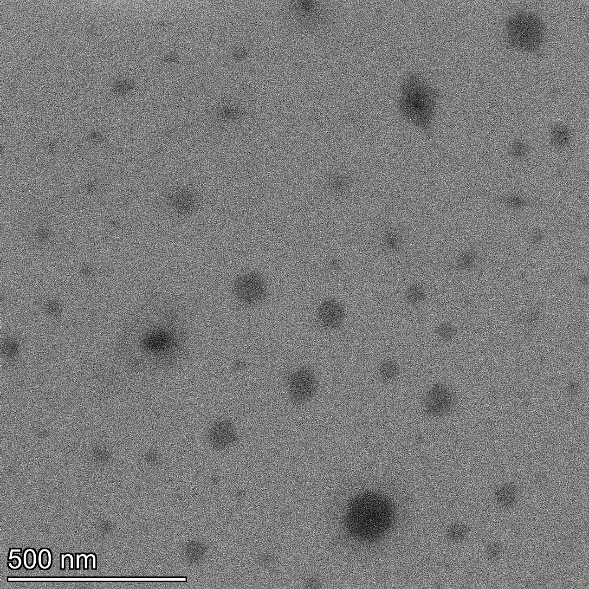


**Figure S12.** Representative TME images of H-LPAE_B4-S4-TMPTA_/DNA polyplexes. Scale bars, 500 nm


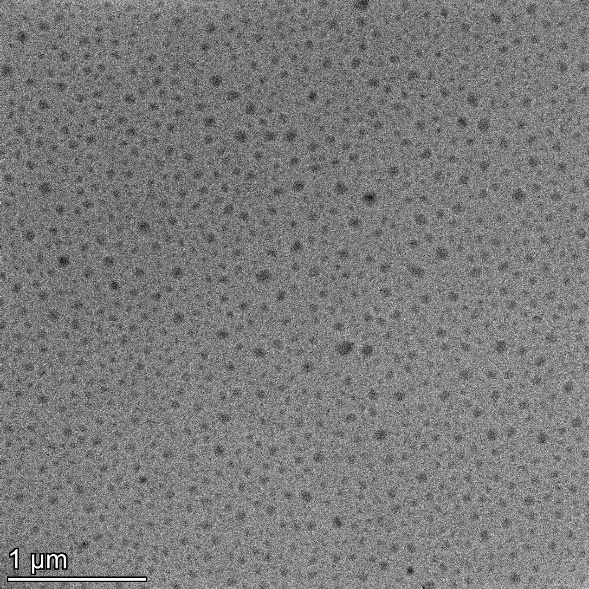


**Figure S13.** Representative TME images of H-LPAE_B4-S4-PET4A_/DNA polyplexes. Scale bars, 1 μm


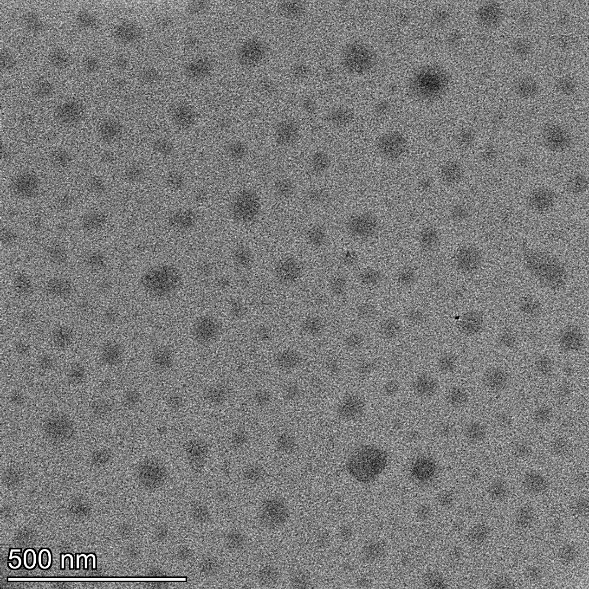


**Figure S14.** Representative TME images of H-LPAE_B4-S4-PET4A_/DNA polyplexes, Scale bars, 500 nm


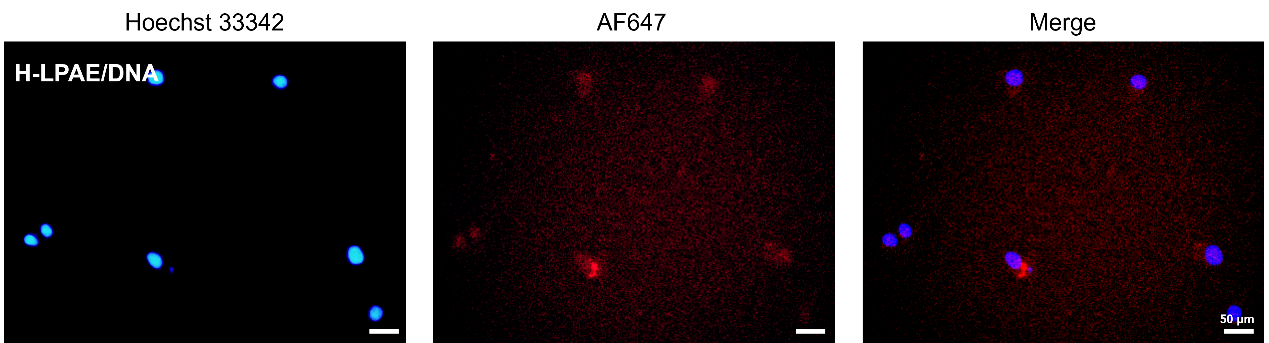


**Figure** **S15.** Fluorescence of cellular uptake with H-LPAE_B4-S5-PET4A_/DNA polyplexes. Scale bars, 50 μm


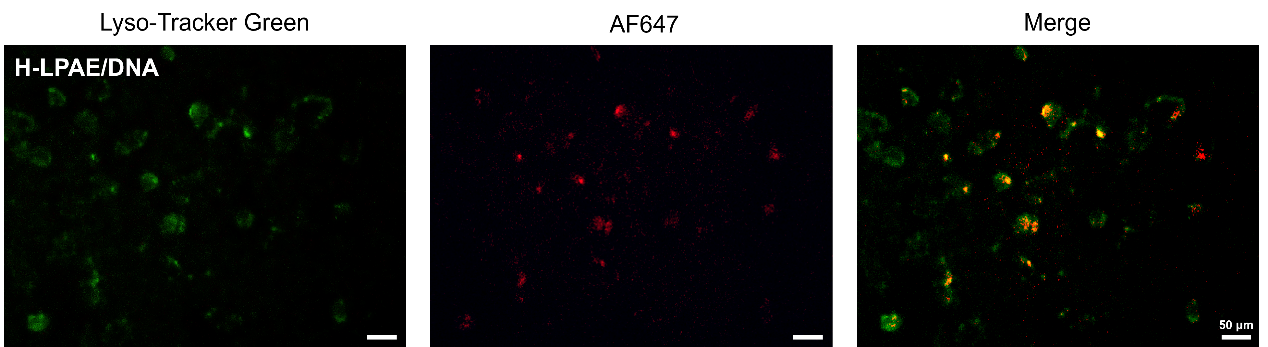


**Figure S16.** Fluorescence of endosomal escape with H-LPAE_B4-S5-PET4A_/DNA polyplexes. Scale bars, 50 μm


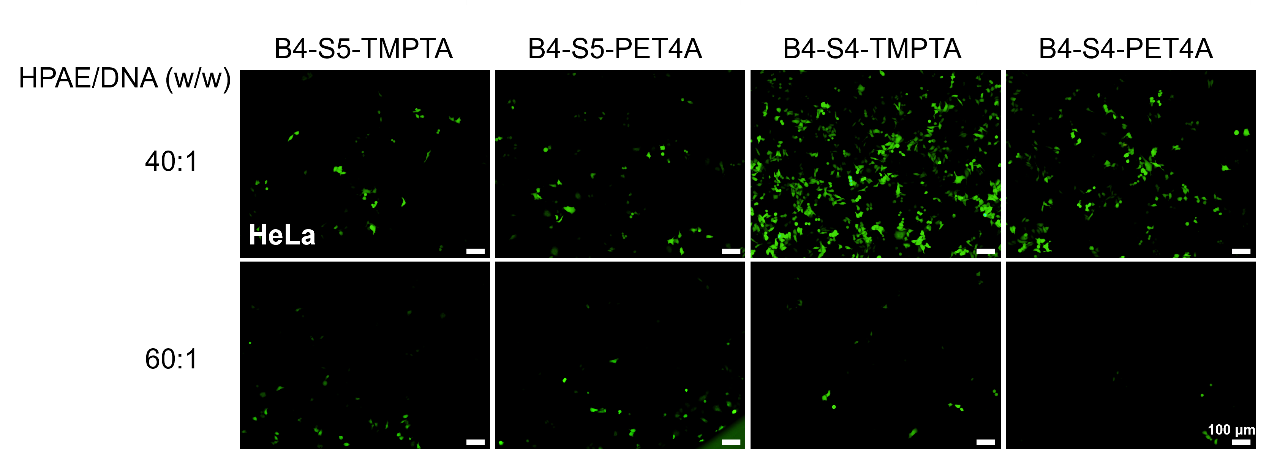


**Figure S17.** Representative GFP images of HeLa after treatment 48 h with various H-LPAEs/DNA polyplexes. Scale bars, 100 μm


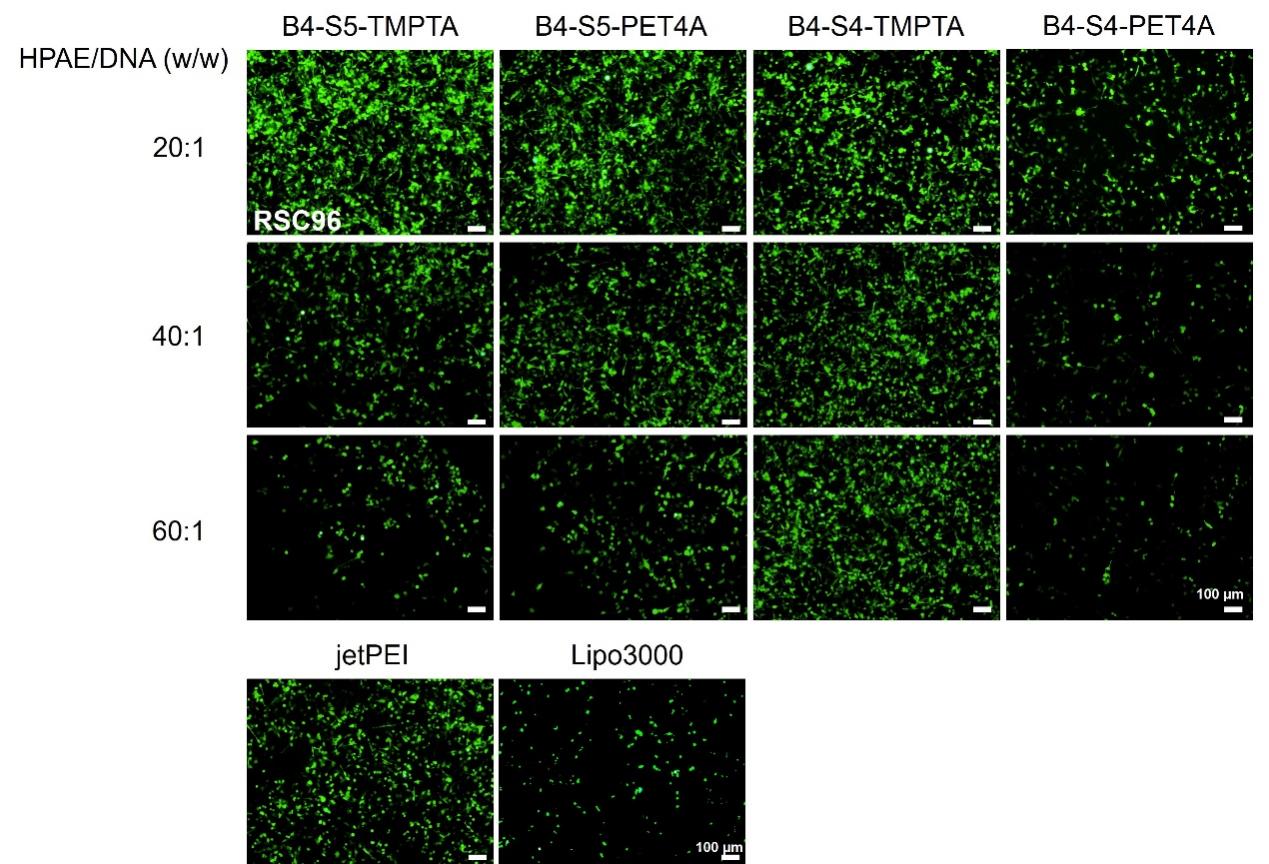


**Figure S18.** Representative GFP images of RSC96 after treatment 48 h with various H-LPAEs/DNA polyplexes. Scale bars, 100 μm


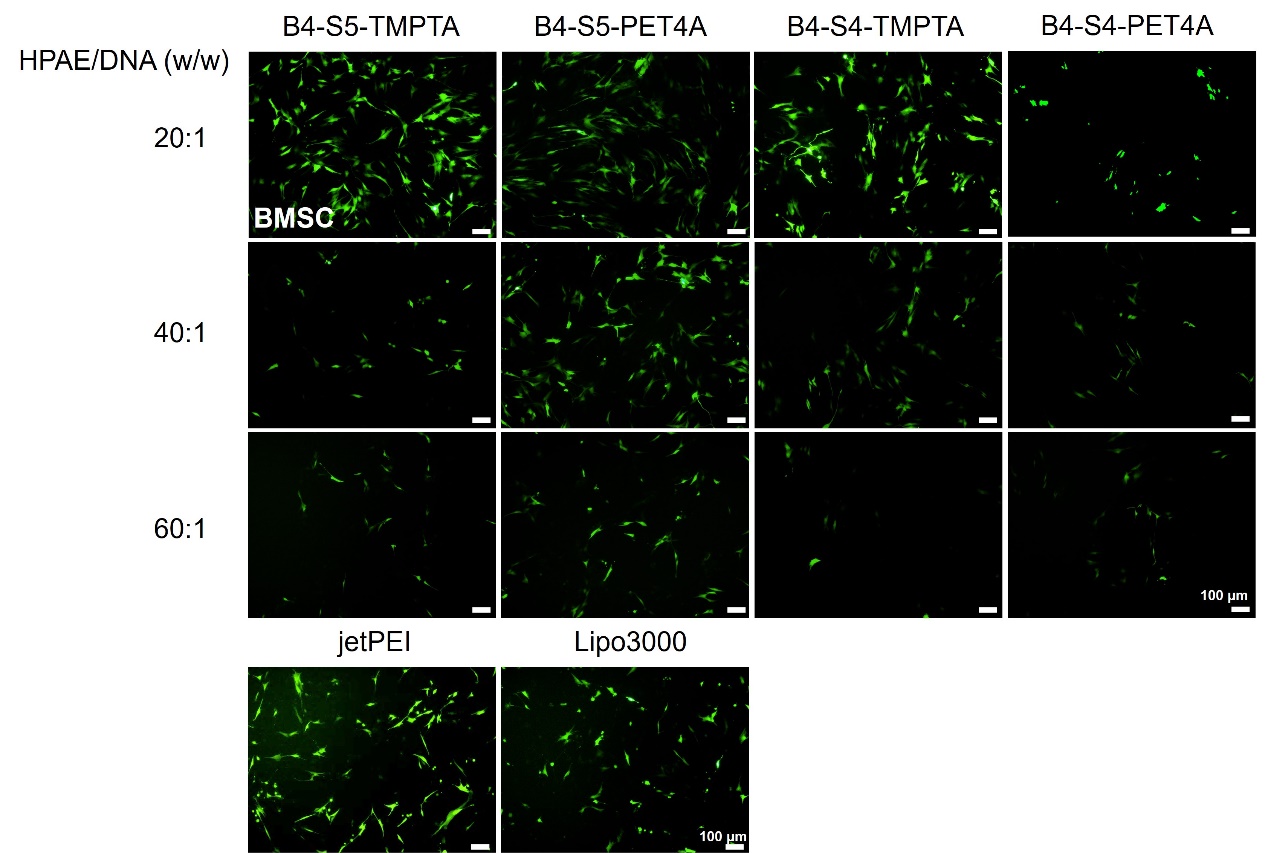


**Figure S19.** Representative GFP images of BMSC after treatment 48 h with various H-LPAEs/DNA polyplexes. Scale bars, 100 μm


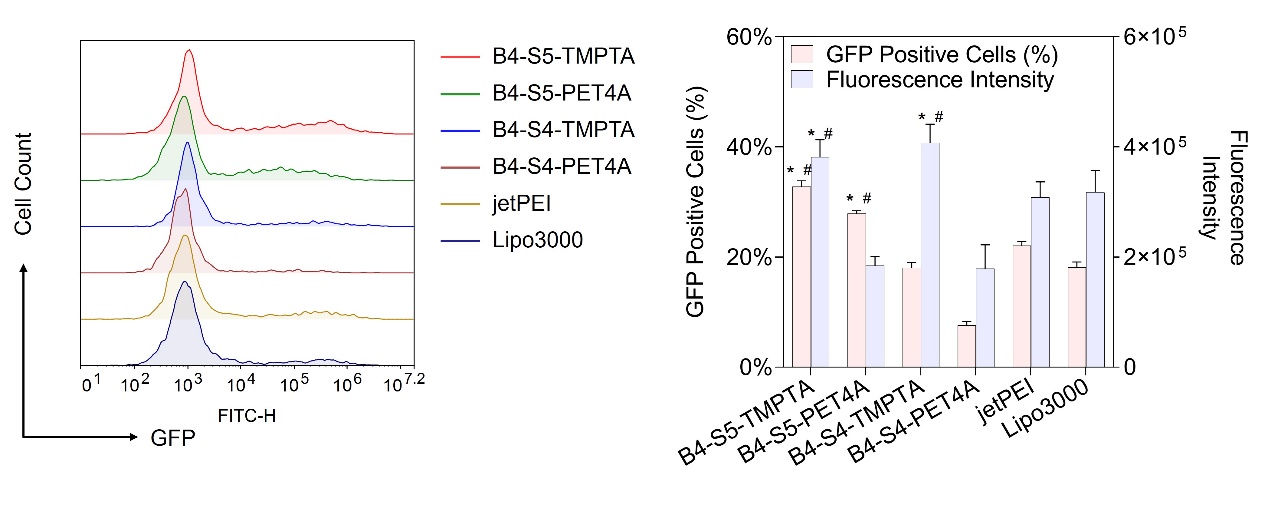


**Figure S20.** Transfection efficiency of H-LPAEs after treatment 48 h in BMSC quantified by flow cytometry. **p* < 0.05 compared to jetPEI, #*p* < 0.05 compared to Lipo3000


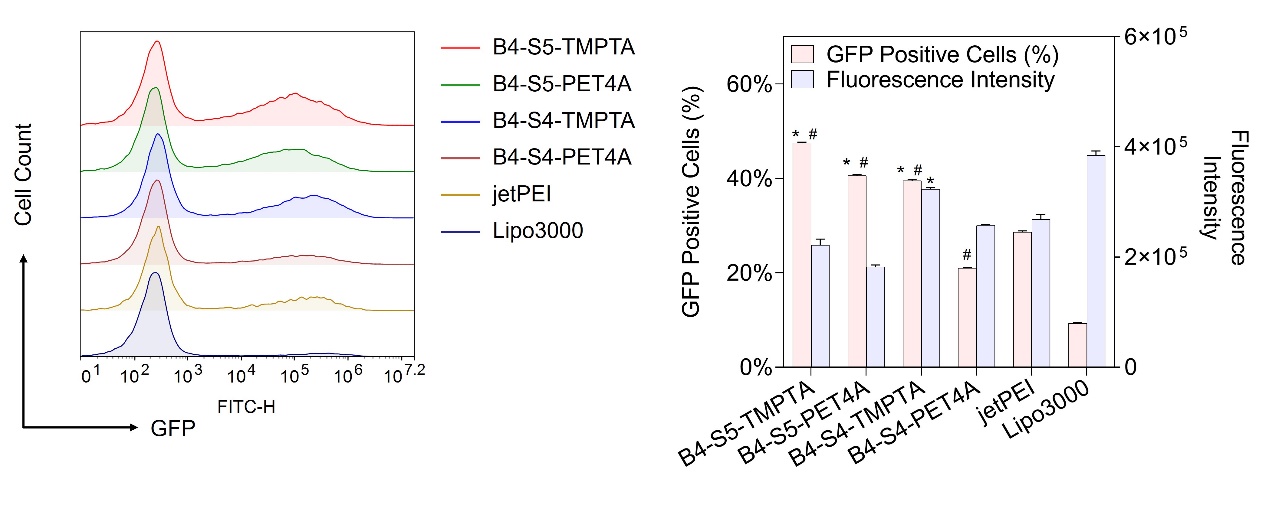


**Figure S21.** Transfection efficiency of H-LPAEs after treatment 48 h and in RSC96 quantified by flow cytometry. **p* < 0.05 compared to jetPEI, #*p* < 0.05 compared to Lipo3000


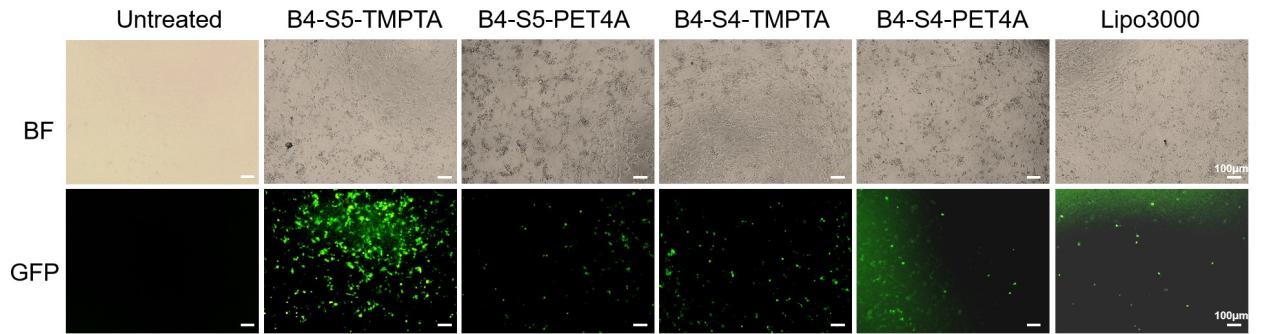


**Figure** **S22.** Representative microscopy images and fluorescence images of SW1353 after transfection 48 h with various H-LPAEs/TRAIL-DNA polyplexes. Scale bars, 100 μm. BF represents bright field.


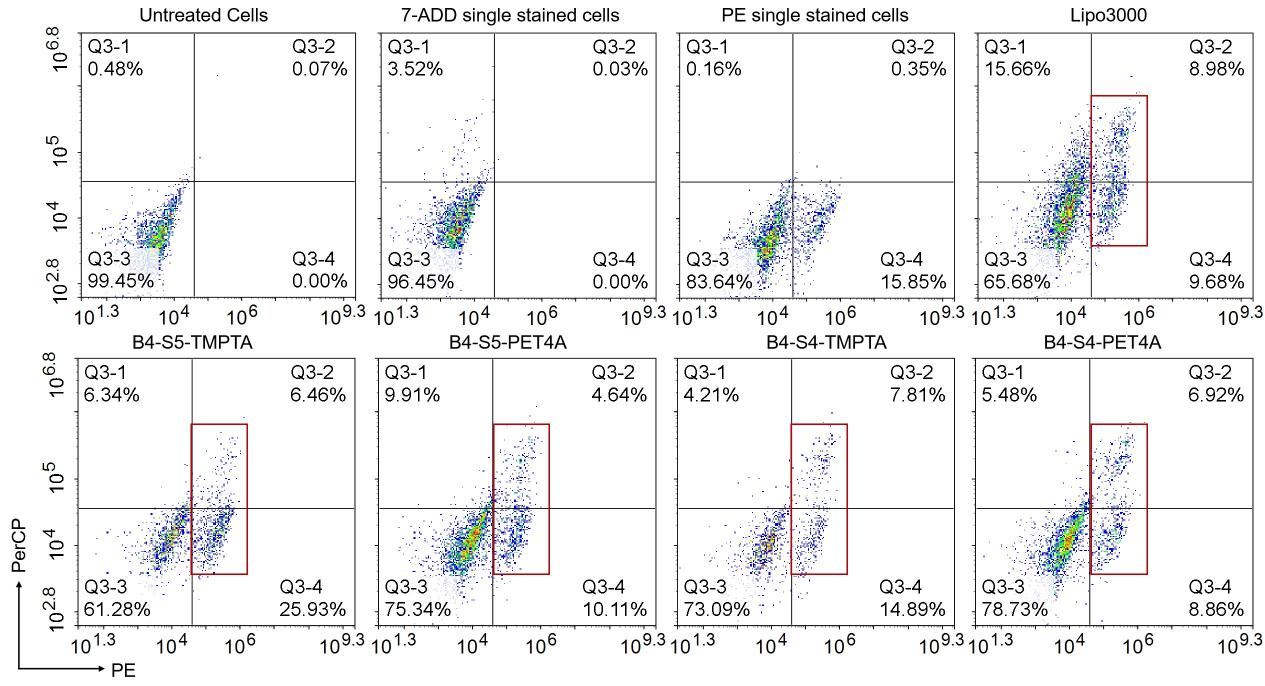


**Figure S23.** Apoptosis efficiency assay of SW1353 after transfection 48 h with various H-LPAEs/DNA polyplexes.


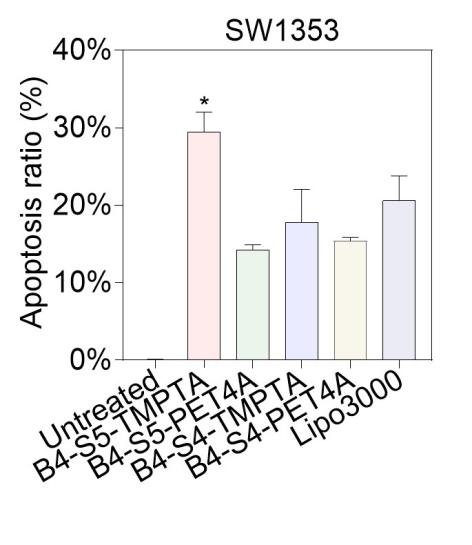


**Figure S24.** Columnar statistical chart of apoptosis efficiency of various H-LPAEs/DNA polyplexes in SW1353 quantified by flow cytometry. **p* < 0.05 compared to Lipo3000
